# Identifying the reactive sites of hydrogen peroxide decomposition and hydroxyl radical formation on chrysotile asbestos surfaces

**DOI:** 10.1186/s12989-019-0333-1

**Published:** 2020-01-20

**Authors:** Martin Walter, Walter D. C. Schenkeveld, Gerald Geroldinger, Lars Gille, Michael Reissner, Stephan M. Kraemer

**Affiliations:** 1 Department of Environmental Geosciences, University of Vienna, Althanstraße 14 (UZA II), 1090 Vienna, Austria; 2 Copernicus Institute of Sustainable Development, Faculty of Geosciences, Utrecht University, Princetonlaan 8A, 3584 CB Utrecht, the Netherlands; 3 Institute of Pharmacology and Toxicology, University of Veterinary Medicine, Veterinärplatz 1, 1210 Vienna, Austria; 4 Institute of Solid State Physics, TU Wien, Wiedner Hauptstraße 8-10, 1040 Vienna, Austria

**Keywords:** Asbestos, Chrysotile, Haber-Weiss, Hydroxyl radical, Fenton, Tetrahedral iron, Mössbauer, EPR

## Abstract

**Background:**

Fibrous chrysotile has been the most commonly applied asbestos mineral in a range of technical applications. However, it is toxic and carcinogenic upon inhalation. The chemical reactivity of chrysotile fiber surfaces contributes to its adverse health effects by catalyzing the formation of highly reactive hydroxyl radicals (HO^•^) from H_2_O_2_. In this Haber-Weiss cycle, Fe on the fiber surface acts as a catalyst: Fe^3+^ decomposes H_2_O_2_ to reductants that reduce surface Fe^3+^ to Fe^2+^, which is back-oxidized by H_2_O_2_ (Fenton-oxidation) to yield HO^•^. Chrysotile contains three structural Fe species: ferrous and ferric octahedral Fe and ferric tetrahedral Fe (Fe^3+^_tet_). Also, external Fe may adsorb or precipitate onto fiber surfaces. The goal of this study was to identify the Fe species on chrysotile surfaces that catalyze H_2_O_2_ decomposition and HO^•^ generation.

**Results:**

We demonstrate that at the physiological pH 7.4 Fe^3+^_tet_ on chrysotile surfaces substantially contributes to H_2_O_2_ decomposition and is the key structural Fe species catalyzing HO^•^ generation. After depleting Fe from fiber surfaces, a remnant fiber-related H_2_O_2_ decomposition mode was identified, which may involve magnetite impurities, remnant Fe or substituted redox-active transition metals other than Fe. Fe (hydr)oxide precipitates on chrysotile surfaces also contributed to H_2_O_2_ decomposition, but were per mole Fe substantially less efficient than surface Fe^3+^_tet_. Fe added to chrysotile fibers increased HO^•^ generation only when it became incorporated and tetrahedrally coordinated into vacancy sites in the Si layer.

**Conclusions:**

Our results suggest that at the physiological pH 7.4, oxidative stress caused by chrysotile fibers largely results from radicals produced in the Haber-Weiss cycle that is catalyzed by Fe^3+^_tet_. The catalytic role of Fe^3+^_tet_ in radical generation may also apply to other pathogenic silicates in which Fe^3+^_tet_ is substituted, e.g. quartz, amphiboles and zeolites. However, even if these pathogenic minerals do not contain Fe, our results suggest that the mere presence of vacancy sites may pose a risk, as incorporation of external Fe into a tetrahedral coordination environment can lead to HO^•^ generation.

## Background

The term asbestos refers to a heterogeneous group of five fibrous amphiboles and one fibrous serpentine mineral (chrysotile) [1, 2]. Due to its favorable properties such as a large tensile strength, heat resistance and non-combustibility, asbestos has been used in a variety of industrial applications [3], e.g. in thermal and electrical insulation, roofing, cement pipes and sheets, flooring and coatings [4, 5]. However, respiratory exposure to asbestos minerals causes adverse health effects like pneumoconiosis, fibrosis of the lung, pleural plaques and effusions, carcinomas predominantly in the lung (but also in the larynx and ovaries) and mesotheliomas in the pleura and peritoneum [2, 4, 6, 7]. Because of their carcinogenic potential, the WHO-IARC has classified all asbestos minerals as group 1 carcinogens [8]. More than 100,000 people die each year because of asbestos-related illnesses, mostly following occupational exposure [9]. Because of the intrinsic health hazard of asbestos, its use has been banned in European countries from the late 1980s onwards [10]. In northern American countries its use has not yet been banned [10] and in some Asian countries it even increases [11, 12].

Chrysotile [Mg_3_Si_2_O_5_(OH)_4_] accounts for more than 95% of all historically used asbestos [13]. As a result, exposure to asbestos predominantly concerns chrysotile. Therefore, we have focused on this mineral in this study. Chrysotile asbestos consists of octahedral Mg hydroxide layers and tetrahedral Si layers which bundle together to a fiber with a Mg hydroxide layer at the surface [14, 15]. During petrogenesis, Fe is substituted into the crystal lattice (usually up to 2–4 wt%) [16]. Ferrous and ferric Fe are found in the Mg layers (Fe^3+^_oct_ and Fe^2+^_oct_, respectively), whereas in the Si layers, exclusively ferric Fe is found (Fe^3+^_tet_) [17, 18]. Fe is by far the most abundant redox-active metal in chrysotile [16].

Weathering of chrysotile at circumneutral pH is commonly described as a layer-by-layer dissolution of alternating Mg and Si layers. Mg layers at the fiber surface dissolve within hours, whereas exposed Si layers dissolve much slower and therefore determine the overall dissolution rate [19, 20]. However, enhanced dissolution of Fe^3+^_tet_ from the Si layer by ligands like the siderophore desferrioxamine-B (DFOB) increases the Si dissolution rate, presumably through the formation of vacancy sites in the Si layer that labilize it [21].

Asbestos-induced pathologies can be linked to its high persistence in vivo [7, 22, 23], its fibrous morphology and the surface chemistry of the fibers [2, 7]. Asbestos fibers lodged in lung or pleural tissue induce continuous, yet unsuccessful attempts of macrophages and neutrophils to phagocytose the fibers - a process called frustrated phagocytosis. During this process, enzymatically formed reactive oxygen species (ROS) like hydrogen peroxide (H_2_O_2_) and superoxide anions (O_2_^•-^) are released into the immediate extracellular environment [7]. Both exhibit a low potency for cellular damage under homeostasis [24] and can be enzymatically detoxified. At elevated concentrations H_2_O_2_ and O_2_^•-^ may, however, interact with Fe on the fiber surface. This interaction induces cyclical redox reactions generating hydroxyl radicals (HO^•^), which have a high potency to damage DNA, proteins and lipids [2, 24–27]. In this Haber-Weiss cycle, Fe acts as a catalyst: Fe^3+^ is reduced by O_2_^•-^ to Fe^2+^, which is back-oxidized by H_2_O_2_ in the so-called Fenton reaction, yielding Fe^3+^ and HO^•^ [2, 28]. In the presence of Fe^3+^, H_2_O_2_ may decompose to hydroperoxyl (HO_2_^•^), which can either directly reduce Fe^3+^ to Fe^2+^ or decompose to the even stronger reductant, O_2_^•-^ [29].

Despite the important role of H_2_O_2_ and its degradation products in Fe redox cycling at the chrysotile fiber surface, H_2_O_2_ decomposition by asbestos has only been assessed in a limited number of studies [30–32]. An involvement of the Fenton and Haber-Weiss pathways in H_2_O_2_ decomposition by asbestos was demonstrated by Eberhardt et al. (1985) [30]. Furthermore, Fubini et al. (1995) [31] assessed H_2_O_2_ decomposition for various Fe containing minerals. They found that H_2_O_2_ decomposition rates by chrysotile and crocidolite were comparable, yet smaller than by magnetite and substantially larger than by hematite.

H_2_O_2_ decomposition by chrysotile partly occurs through Fenton reactions involving Fe surface species [2, 25, 33]. However, not all Fe surface species are equally Fenton-active or have an equal potential to form hydroxyl radicals. Fubini et al. (1995) [31] demonstrated that Fe^2+^_oct_ on chrysotile surfaces does not play a substantial role in HO^•^ generation. Recently, Walter et al. (2019) suggested that per mole Fe the potential to generate HO^•^ is substantially larger for surface exposed Fe^3+^_tet_ than for Fe_oct_ [21]. Furthermore, Fe^3+^_tet_ is the only Fe surface species in chrysotile that remains Fenton-active during long-term dissolution (weeks) at circumneutral pH, because the Si layer in which it is incorporated dissolves slowly, whereas Fe_oct_ in the readily dissolving Mg layers rapidly precipitates to Fenton-inactive Fe (hydr)oxide minerals [21]. Depletion of all Fe surface species (including Fe^3+^_tet_) from chrysotile surfaces by ligands like DFOB decreased the radical yield of the fibers, almost to background values [21, 33]. Apart from structural Fe, also external Fe that associates with surfaces of asbestos (or other silicates) may generate ROS and increase oxidative stress in vivo and in vitro [2, 34–37].

To our knowledge, the relation between Fe speciation at chrysotile fiber surfaces and H_2_O_2_ decomposition rates has not yet been established. Also, the relation between the speciation of external Fe after associating with the chrysotile fiber surface and the change in radical yield and H_2_O_2_ decomposition rate of the fibers has not been explored previously. Hence, the current understanding of which Fe species at the chrysotile surface participate in the prerequisite step of the first stage (H_2_O_2_ decomposition to reductants), and in the second stage (Fenton oxidation) of the Haber-Weiss cycle is incomplete. Establishing the reactive sites of H_2_O_2_ decomposition and HO^•^ generation on chrysotile surfaces is important in assessing the overall redox reactivity of chrysotile asbestos, which is a major determinant in its pathogenicity [25, 38]. In this study we addressed this knowledge gap.

We hypothesize that H_2_O_2_ is decomposed, either by structural Fe^3+^_tet_ in exposed Si layers of the dissolving fibers, or by secondary Fe minerals precipitated on the fiber surface. The precipitated Fe may originate from external sources or from fiber dissolution during which structural Fe is released. Furthermore, we hypothesize that external Fe only substantially contributes to the HO^•^ yield of chrysotile fibers when it becomes tetrahedrally coordinated by incorporation into a Si layer. The rationale for this hypothesis is the high potential of surface Fe^3+^_tet_ for generating HO^•^ [21], compared to the low potential of Fe (hydr)oxides [37] precipitated on chrysotile surfaces. Finally, we hypothesize that chrysotile fibers with surfaces depleted in Fe (e.g. due to preconditioning with a ligand) may still pose a health hazard if external Fe is incorporated into vacant surface sites in the Si layer.

The hypotheses were tested in batch incubation experiments. Samples were analyzed by ICP-OES (inductively coupled plasma optical emission spectrometry), UV-VIS-photospectrometry, Mössbauer spectroscopy and EPR (electron paramagnetic resonance) spectroscopy.

## Methods

### Chemical reagents and asbestos characterization

All chemical reagents used in this study were at least pro analysis grade and were ordered from VWR (unless otherwise mentioned). Chrysotile asbestos was purchased from Shijiazhuang Mining IMP&EXP Trade Co, China. The material was characterized by XRD-Rietveld phase analysis, Raman spectroscopy, BET specific surface area measurement, Mössbauer spectroscopy, fusion digestion and neutron activation analysis [21]. The BET specific surface area (SSA) of Shijiazhuang chrysotile fibers was 20.3 m^2^ g^− 1^ (with a standard deviation of 0.9 m^2^ g^− 1^, [21]), and phase impurities were established by XRD-Rietveld analysis: Shijiazhuang chrysotile contains 86.4 ± 4.6% chrysotile fibers, whereas phase impurities in the fiber material are brucite, talc, chlorite, magnetite, quartz and calcite [21]. Key results on the bulk of Shijiazhuang chrysotile are presented in Table 1: Shijiazhuang chrysotile asbestos contains ≈249 g kg^− 1^ Mg and ≈188 g kg^− 1^ Si; the stoichiometric Mg/Si ratio is close to 1.5. Fe (≈20 g kg^− 1^) and Al (≈8 g kg^− 1^) are the major substituents. Mössbauer analyses demonstrated that in pristine Shijiazhuang chrysotile asbestos, almost all Fe is substituted into the octahedral Mg layer (≈ 55% Fe^3+^_oct_ and ≈ 38% Fe^2+^_oct_), whereas only 7% is substituted into the tetrahedral Si layer (Table 1). Magnetite (1.5 ± 0.2% in Shijiazhuang chrysotile) hosts approximately 32% of the total bulk Fe (Table 1).

**Table 1:** Bulk characteristics of pristine Shijiazhuang chrysotile asbestos (previously reported in Walter et al. (2019) [21]). Values in round brackets represent standard deviations

Bulk characteristics of chrysotile asbestos			
Bulk composition:		Fusion digestion (*n* = 15):	NAA^a^ (*n* = 2):
Mg	[g kg^−1^]	249 (7)	
Si	[g kg^−1^]	188 (3)	
Fe	[g kg^−1^]	19.0 (1.4)	21.4 (0.3)
Al	[g kg^−1^]	8.0 (0.5)	
Bulk Fe speciation:		*Mössbauer:*	
Fe^2+^_oct_	[%]	38.4	
Fe^3+^_oct_	[%]	54.6	
Fe^3+^_tet_	[%]	7.0	
Total Fe in chrysotile^b^	[%]	68.2	

^a^Neutron activation analysis

^b^Remaining Fe (31.8%) is in magnetite impurities

### Preparation of fiber suspensions

All experiments were carried out in fiber suspensions with a fiber to solution ratio of 1 g L^− 1^. The non-metal-complexing tertiary amine (“Better”) buffer [39] MOPS (3-(*N*-morpholino)propanesulfonic-acid) was used at a concentration of 50 mmol L^− 1^ to maintain the pH of experimental solutions at 7.4 ± 0.3. The ionic strength of the buffer solutions was adjusted to 300 mmol L^− 1^ by addition of NaCl. Solutions in blank treatments contained only pH-buffer and electrolyte, while DFOB (Novartis) treatments additionally contained 1 mmol L^− 1^ DFOB. In H_2_O_2_ decomposition experiments DFOB was used to quench the redox-activity of Fe. This method has been used previously, e.g. in refs [40, 41]. Finally, H_2_O_2_ decomposition was also studied in 0.1 mol L^− 1^ NaOH solutions in which chrysotile fibers are practically insoluble [21].

### Preconditioning of chrysotile fibers

Fibers were preconditioned to obtain fiber types with different specific surface chemistry. The preconditioning involved incubation of the fibers in blank solutions buffered at pH 7.4 for 336 h (“blank-altered fibers”) or in 1 mmol L^− 1^ DFOB solutions buffered at pH 7.4 (“DFOB-altered fibers”). In previous studies it was shown that in blank-altered fibers, the outermost Mg layer had dissolved during preconditioning and the Fe content of the dissolved Mg layer had precipitated as secondary Fe phases with low Fenton activity [21, 37]. Moreover, in DFOB-altered fibers the Fe content of the dissolved outermost Mg layer as well as the Fe content of the slowly dissolving Si layer was complexed and mobilized by DFOB. Fe mobilization from the Si layer presumably leads to the formation of vacancy sites, which promote Si dissolution [21]. During preconditioning up to 4% of the fiber mass dissolved; assuming a cylindrical fiber geometry with constant length, this corresponds with a 2% decrease in SSA, which is smaller than the standard deviation on the BET-SSA analysis and was considered negligible.

**Fig. 1:**
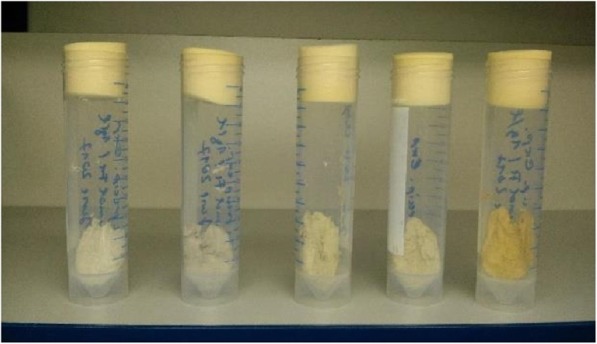
Preconditioned fibers. From left to right: DFOB-altered fibers with 0, 3 and 30 μmol g^− 1^ Fe, respectively, blank-altered fibers with 0 μmol g^− 1^ Fe and DFOB-altered fibers with 300 μmol g^− 1^ Fe

To test whether external Fe can be incorporated into vacancy sites in the Si layer and whether this incorporated Fe participates in H_2_O_2_ decomposition and HO^•^ generation, DFOB-altered fibers were suspended in solutions buffered at pH 7.4 containing 0, 3, 30 and 300 μmol L^− 1^ of Fe^2+^ under anoxic conditions in a N_2_-filled anoxic chamber (Brown box). The suspensions were then immediately oxygenated outside the anoxic chamber by air bubbling for 24 h, while magnetically stirring them at 500 rotations per minute. The Fe^2+^ rapidly oxidized and Fe not incorporated into vacancy sites precipitated onto fiber surfaces as Fe (hydr)oxide minerals, coloring the fibers beige to yellow (see Fig. 1). As a negative control, the same concentrations of Fe were precipitated onto blank-altered fibers (which presumably lack vacancy sites in the Si layer) following the same procedure. The obtained altered fiber types are referred to as “DFOB-altered fibers + 0, 3, 30 or 300 μmol g^− 1^ Fe” and “blank-altered fibers + 0, 3, 30 or 300 μmol g^− 1^ Fe”. Preconditioned fibers were collected in Büchner funnels on 0.47 μm Nylon membranes (Magna) and dried by vacuum filtration. To remove potentially adsorbed DFOB ligand or metal-DFOB complexes, fibers were washed with ultra-pure water and then vacuum-dried and stored in an evacuated desiccator until they were used in follow-up experiments. Metal and Si concentrations mobilized during the fiber preparations are presented in Additional file 1: Table S1.

### ^57^Fe addition and Mössbauer analyses

^57^Fe Mössbauer spectroscopy was performed at room temperature in standard constant acceleration mode with a ^57^Co**Rh** source, relative to which all center shift data are given. The analyzed fiber types were DFOB-altered and blank-altered fibers + 0 μmol g^− 1^ Fe, and DFOB-altered and blank-altered fibers + 3 μmol g^− 1^ Fe. These fiber types were prepared following the procedure described above, except that isotopically enriched ^57^Fe (Sigma Aldrich, > 95 atom % isotopic purity) was used. The isotopically enriched metallic ^57^Fe-powder was dissolved over night at 70 °C in a 2 mol L^− 1^ HCl solution, according to Arrigo et al. (2017) [42]. This procedure yielded a ^57^Fe^2+^ solution, which was purged with N_2_ for 2 h and then put into the anoxic glove box. The isotopic composition of Fe in the stock solution was verified by ICP-MS (^57^Fe accounted for 99.2% of the total Fe), and the Fe^2+^ concentration was verified spectrophotometrically with a ferrozine assay [43]. Aliquots of the ^57^Fe^2+^ stock solution were added to DFOB-altered and blank-altered fiber suspensions to obtain an added concentration of 3 μmol g^− 1 57^Fe^2+^.

**Fig. 2:**
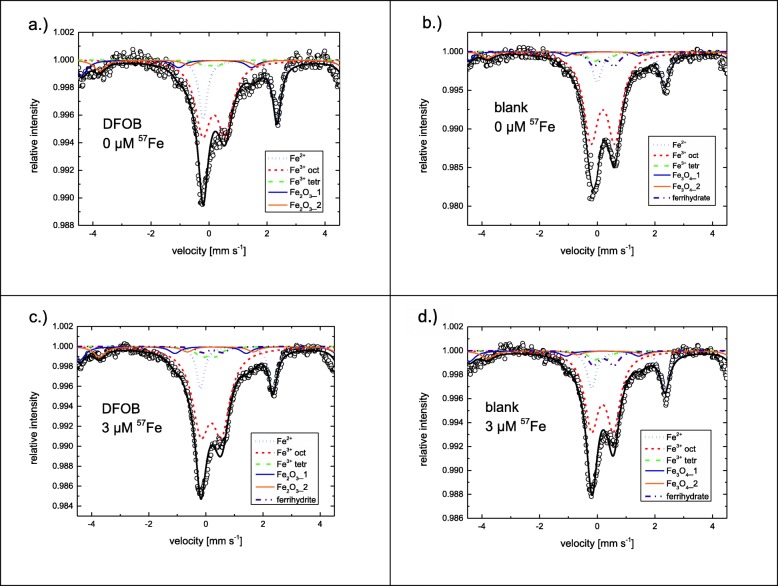
Narrow velocity range Mössbauer spectra of DFOB-altered fibers (Panel a and c) and blank-altered fibers (Panel b and d) with 0 or 3 μmol g^− 1^ added ^57^Fe. Spectra for blank-altered fibers and DFOB-altered fibers + 3 μmol g^− 1 57^Fe were fitted with a ferrihydrite sub-spectrum to account for Fe (hydr)oxide precipitation

After vacuum filtration and drying of the fibers, 700 mg of each fiber type were ground in a tungsten carbide ball mill (Resch Schwingmühle MM 400) for 30 s (a duration that does not affect Fe^2+^/Fe^3+^ ratios in minerals [44]) at 30 strokes per minute in order to avoid spatial anisotropy of fibers in specimens. 500 mg of the milled fibers were pressed between Teflon foils (Zuma). Mössbauer measurements required up to 2 weeks per samples (Fig. 2). The spectroscopic data were analyzed by solving the full Hamiltonian. Thickness of the samples was taken into account after Mørup and Both (1975) [45]. A ferrihydrite sub-spectrum (based on data from Murad and Schwertmann, 1980, [46]) was used to account for Fe precipitation on blank-altered fibers + 0 μmol g^− 1 57^Fe (precipitation of Fe from the dissolved Mg layer) and DFOB-altered and blank-altered fibers + 3 μmol g^− 1 57^Fe (precipitation of added ^57^Fe). Ferrihydrite was selected, because under the experimental conditions such a poorly crystalline Fe^3+^ (hydr)oxide mineral is most likely to precipitate. Fits involving DFOB-altered fibers + 0 μmol g^− 1 57^Fe were done with and without ferrihydrite sub-spectrum; including the ferrihydrite sub-spectrum did not significantly improve the fit. Because presumably precipitation of ferrihydrite was prevented by addition of DFOB, the fit without the ferrihydrite sub-spectrum was used for comparison with the other treatments. Each sample was measured two times: first in a wider velocity range (± 10.6 mm s^− 1^) to cover the full magnetically split-spectrum of magnetite impurities, which allowed to obtain the amount of magnetite in the samples, and second in a narrow velocity range (± 4.6 mm s^− 1^) to better resolve the chrysotile and Fe^3+^ (hydr)oxide contributions. The obtained hyperfine parameters for both velocity ranges are presented in Additional file 1: Table S2, the spectra of the narrow velocity range are presented in Fig. 2 and the spectra of the wide velocity range in Additional file 1: Figure S1. The magnetite contents were calculated based on the wide velocity range data. After determination of the percentage of magnetite, the percentages of remaining Fe species were determined using the narrow velocity range data by multiplying the narrow velocity range percentages of these Fe species with (100% - magnetite% (wvr))/(100% - magnetite% (nvr)). Finally, all percentages were multiplied by the total amount of ^57^Fe in each treatment. For blank-altered fibers the Fe content equaled the average content in pristine fibers measured by neutron activation analysis (NAA, Table 1), the amount of Fe removed by DFOB in DFOB-altered fibers was determined from the dissolved Fe concentration after reaction with DFOB. The amount of ^57^Fe added was known.

### Experimental procedure for H_2_O_2_ decomposition experiments

In the H_2_O_2_ decomposition experiments, metal mobilization from, and decomposition of H_2_O_2_ by, differently preconditioned fibers were assessed. Included fiber types were: pristine fibers, blank-altered fibers, DFOB-altered fibers and both blank-altered and DFOB-altered fibers + 0, 3, 30 or 300 μmol g^− 1^ Fe. Experiments were carried out at pH 7.4 in MOPS buffer, and additionally for pristine and blank-altered fibers in a 0.1 mol L^− 1^ NaOH solution. The initial experimental H_2_O_2_ concentration was 3.3 g L^− 1^ (≈ 0.3%), which was prepared by diluting a 30% stock solution (Sigma Aldrich, for trace analysis) a hundred times. The H_2_O_2_ concentration of the stock was determined by redox titration with KMnO_4_: 334 ± 2 g L^− 1^ H_2_O_2_. Experiments were carried out in duplicates in 15 ml PP tubes (VWR) that were shaken in an end-over-end shaker at 15 rounds per minutes (RPM) at 20 ± 2 °C in the dark. Samples were taken destructively after 0.5, 1, 4, 8, 24, 48, 96, 168 and 336 h. Suspensions were filtered over 0.45 μm Sartorius cellulose acetate syringe filters. An aliquot of each filtrate was acidified to 0.14 mol L^− 1^ HNO_3_ (trace metal grade) for metal (Mg and Fe) and Si concentration analysis by ICP-OES (Perkin Elmer Optima 5300-DV). Another aliquot of each filtrate was diluted for H_2_O_2_ concentration measurements. Calibration standards for ICP-OES analysis were matrix-matched with the samples. The decomposition of H_2_O_2_ was assessed by measuring the H_2_O_2_ concentration in diluted filtrates immediately after each sampling round. H_2_O_2_ concentrations were determined spectrophotometrically by a titanium sulfate method [47]. One ml of a 1.9–2.1% titanium (IV) oxysulfate solution (Sigma Aldrich) was added to 0.5 ml of the diluted filtrate and light absorption by the resulting peroxytitanyl-ion was measured at 410 nm by a Varian Cary 50 UV/VIS spectrophotometer (ɛ = 689 L mol^− 1^ cm^− 1^). H_2_O_2_ concentrations in the samples were quantified by an external linear calibration method (7 to 42 mg L^− 1^ H_2_O_2_); filtrates were diluted down to fit the calibration range. Because H_2_O_2_ also reacts with MOPS buffer [48], a control treatment to determine the H_2_O_2_ decomposition rate in absence of fibers was also included. Also for experiments in 0.1 mol L^− 1^ NaOH a control treatment without fibers was included. In an additional experiment, H_2_O_2_ decomposition by pristine, blank-altered and DFOB-altered fibers was examined at pH 7.4 in the presence of 1 mmol L^− 1^ DFOB using the same experimental procedure. The absorption maximum of the FeDFOB complex (425 nm; ɛ = 2460 L mol^− 1^ cm^− 1^, [49]) and the peroxytitanyl-ion (vide supra) [47, 50] are in close proximity. However, FeDFOB concentrations were orders of magnitude smaller and the molar absorption coefficients of the complexes are less than one order of magnitude different. Therefore, the contribution of FeDFOB to overall light absorption at 410 nm could be neglected.

### EPR spin trapping analyses of hydroxyl radicals generated by Fe on chrysotile fibers surfaces

The HO^•^ yield of fiber specimens in the presence of H_2_O_2_ was quantified with 5–5-dimethyl-1-pyrroline N-oxide (DMPO) as spin trapping agent using a X-band EPR-spectrometer (Bruker EMX) and a split ring resonator (Bruker MD5). This spin trapping technique has frequently been used for this purpose before [26, 31, 37, 51, 52]. Eleven mg of fibers were incubated for 0.5 h in 0.5 ml of a 125 mmol L^− 1^ H_2_O_2_ and 12.5 mmol L^− 1^ DMPO solution buffered at pH 7.3 with a 250 mmol L^− 1^ chelex-treated phosphate buffer. After 25 min of incubation at room temperature and 5 min of centrifugation (14,000 RPM), 50 μl of the supernatant were pipetted into a glass capillary (intraMark Blaubrand), which was then sealed with Critoseal. Subsequently, the capillary was transferred into the resonator. The instrumental settings for the EPR measurements are described in Walter et al. (2019) [21]. EPR measurements were performed on four subsamples from each type of preconditioned fibers (quadruplicates). To quantify the change in HO^•^ yield, the signal intensity (Intensity peak-to-peak (Ipp)) of the second peak from the left in the DMPO/HO^•^ quadruplet of altered fibers was determined and expressed as a percentage of the Ipp of pristine fibers, which was measured as a reference in each measurement session. For comparison, also the HO^•^ yield of the poorly crystalline Fe (oxy)hydroxide 2-line ferrihydrite (3 ± 0.2 mg, synthesized according to Schwertmann and Cornell (2000), [53]) was measured following the same procedure. An amorphous Fe (hydr)oxide like 2-line ferrihydrite may precipitate upon Fe addition to the fibers and subsequent oxygenation [53, 54].

### Statistical analysis and supplementary data

Statistical analysis of the EPR spin trapping data was performed with the program SPSS Version 25. A square root transformation of the data was carried out to reduce skewness. Homogeneity of the transformed data was tested with the Levene’s test (α = 0.05). Differences among treatments were established by applying the univariate general linear model procedure and the Tukey post-hoc test (α = 0.05). A statistical test was employed to answer a) if the HO^•^ yield increased with the amount of Fe applied to DFOB-altered fibers and b) if, through addition of Fe to DFOB-altered fibers, the HO^•^ yield of blank-altered fibers could be reached.

**Fig. 3:**
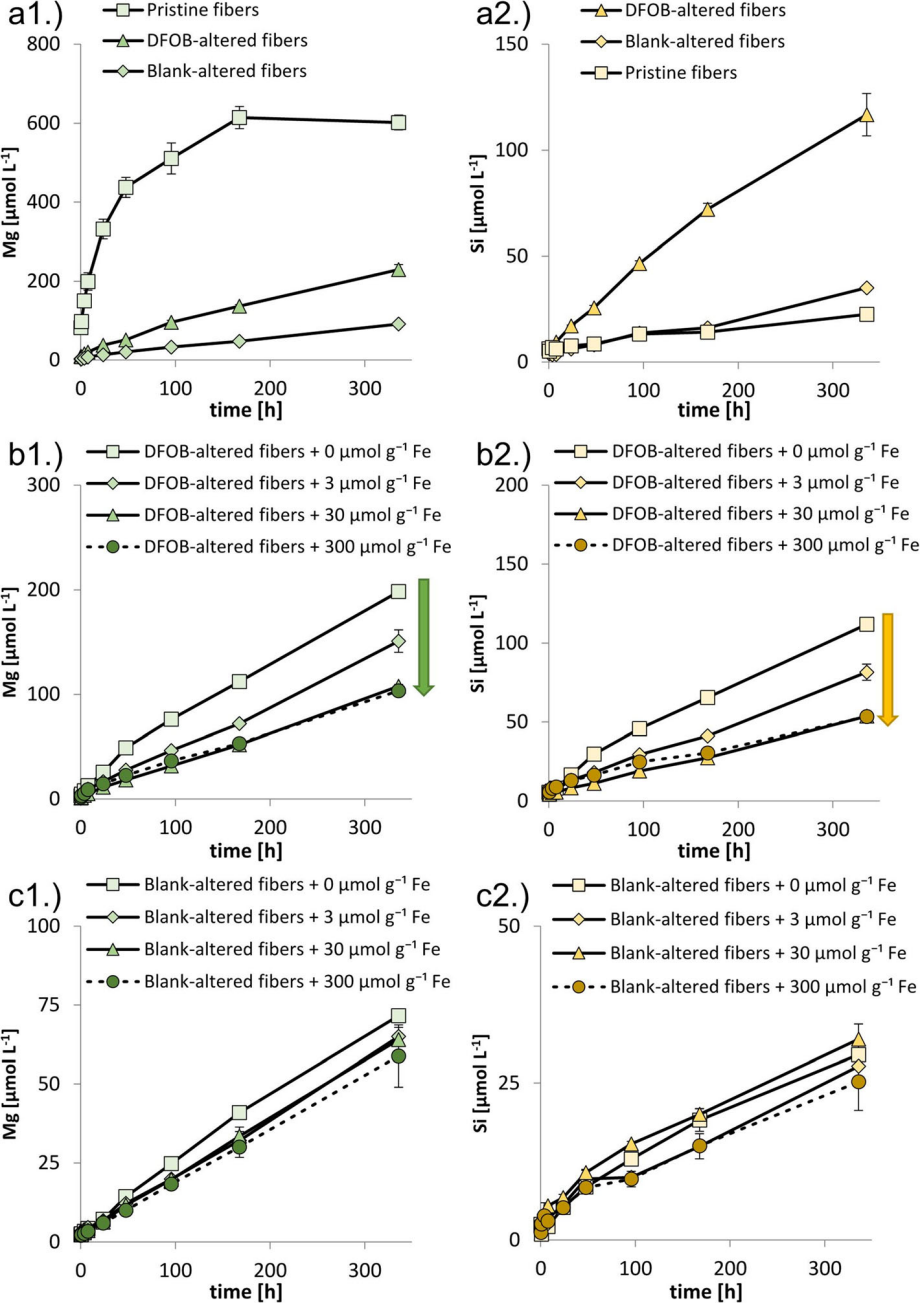
Mg and Si mobilization from 1 g L^− 1^ pristine and preconditioned fibers incubated at pH 7.4 (50 mmol L^− 1^ MOPS) with addition of 3.34 g L^− 1^ H_2_O_2_. Panel a.) Mobilized Mg (a1) and Si (a2) concentrations from pristine, blank-altered and DFOB-altered fibers; Panel b.) Mobilized Mg (b1) and Si (b2) concentrations from DFOB-altered fibers + 0, 3, 30 and 300 μmol g^− 1^ Fe. The arrows indicate a decrease in mobilized Mg and Si concentration with increasing Fe addition; Panel c.) Mobilized Mg (c1) and Si (c2) concentrations from blank-altered fibers + 0, 3, 30 and 300 μmol g^− 1^ Fe. Error bars indicate standard deviations (*n* = 2)

**Fig. 4:**
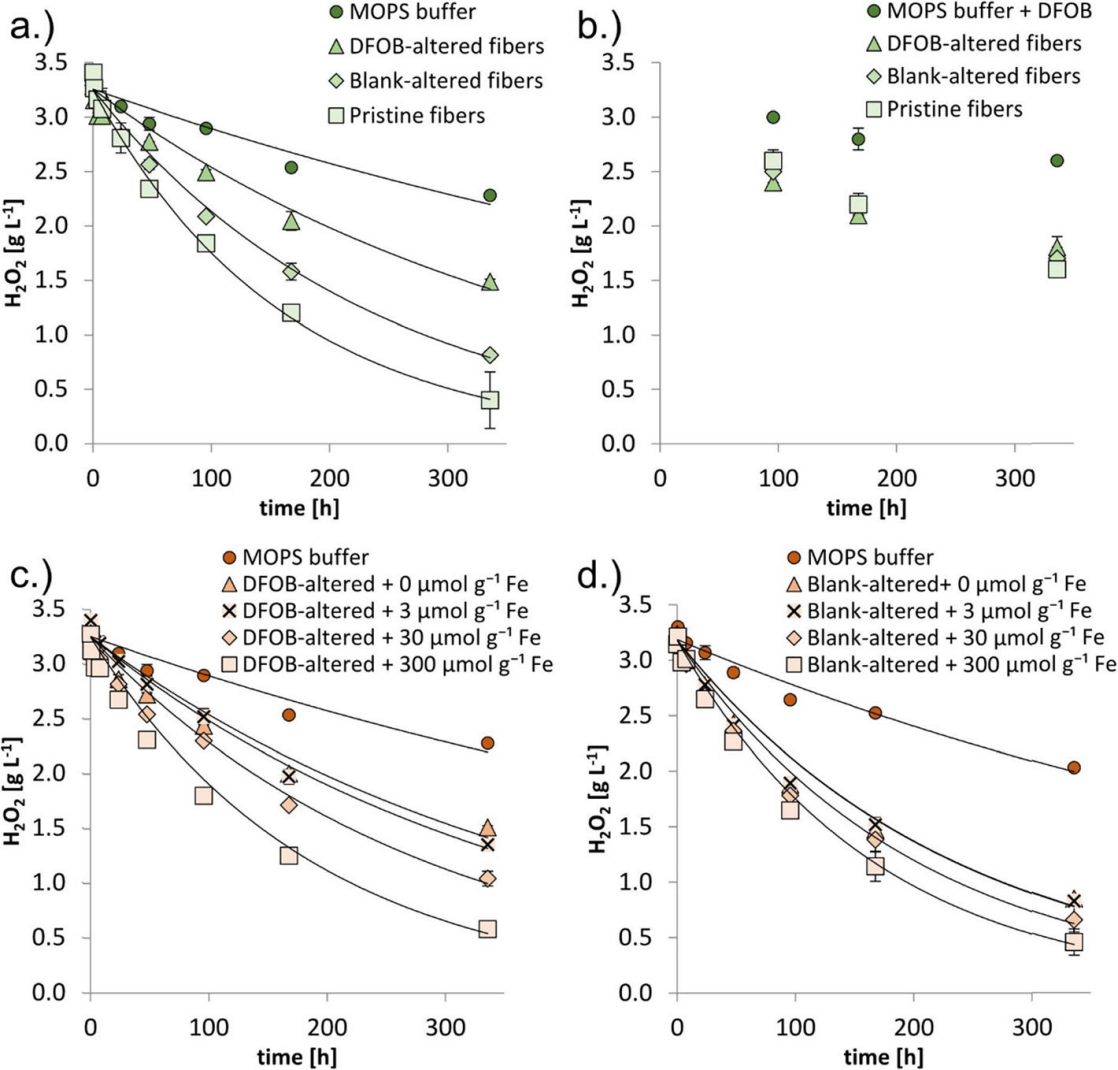
Decomposition of H_2_O_2_ (initial concentration 3.34 g L^− 1^) by 1 g L^− 1^ pristine and preconditioned chrysotile fibers at pH 7.4 (50 mmol L^− 1^ MOPS). Parameters of the exponential fits of the H_2_O_2_ concentration data are presented in Table 2. Panel a.) Decomposition of H_2_O_2_ in the presence of pristine, blank-altered and DFOB-altered fibers; Panel b.) H_2_O_2_ decomposition in the presence of MOPS buffer + 1 mmol L^− 1^ DFOB, in absence of fibers, and in presence of pristine, blank-altered and DFOB-altered fibers; Panel c-d.) Decomposition of H_2_O_2_ in absence of fibers and in presence of DFOB-altered fibers + 0, 3, 30 and 300 μmol g^− 1^ Fe (Panel c) and blank-altered fibers + 0, 3, 30 and 300 μmol g^− 1^ Fe (Panel d). Error bars indicate standard deviations (*n* = 2)

**Fig. 5:**
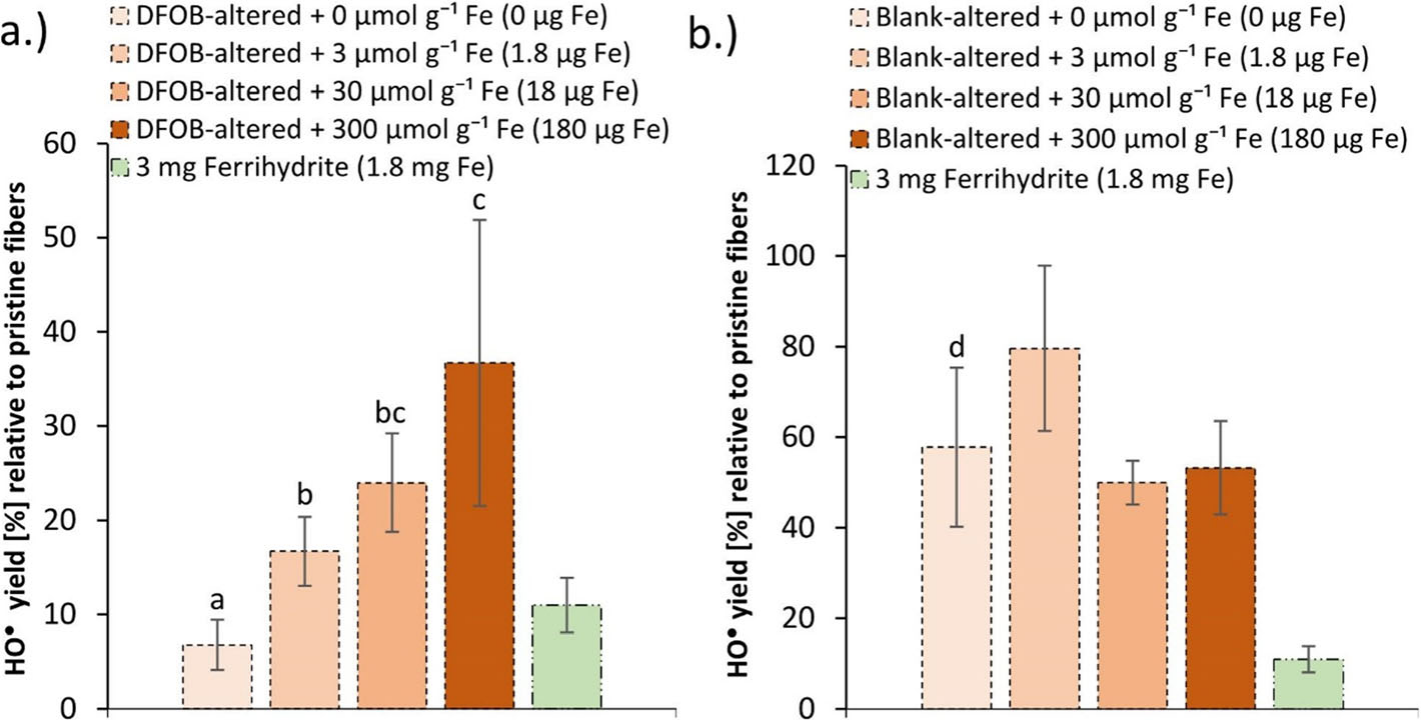
HO^•^ yield of preconditioned fibers and ferrithydrite measured by the DMPO/HO^•^-EPR signal intensity. The signal is expressed as a precentage of the HO^•^ yield of pristine fibers, which was analyzed at every measuring session as a reference. Panel a.) HO^•^ yield of DFOB-altered fibers + 0, 3, 30 and 300 μmol g^−1^ Fe, and 3 mg ferrihydrite; Panel b.) HO^•^ yield of blank-altered fibers + 0, 3, 30 and 300 μmol g^− 1^ Fe and 3 mg ferrihydrite. The letters indicate significantly different HO^•^ yields as identified by the Tukey post-hoc test following an ANOVA. The results illustrate an increase in HO^•^ yield upon addition of Fe to DFOB-altered fibers, yet the HO^•^ yield of blank-altered fibers was not reached. Error bars indicate standard deviations (*n* = 4)

The data included in Figs. 2, 3, 4 and 5 are reported in Additional file 1: Table S2 to Table S5, respectively. The *p*-values from the statistical analyses of the EPR data are reported in Additional file 1: Table S6.

## Results

### Color changes related to Fe at chrysotile surfaces

Complexation and mobilization of Fe from the beige pristine chrysotile fibers by DFOB resulted in the whitish color of DFOB-altered fibers (Fig. 1). Interaction of DFOB-altered fibers with 3 μmol g^− 1^ Fe changed the whitish color to greyish after exposure to oxygen; interaction with 30 μmol g^− 1^ Fe reversed the color to beige, comparable to the color of pristine (not shown) and blank-altered fibers, and interaction with 300 μmol g^− 1^ Fe changed the color to yellow (Fig. 1). Interaction of pristine fibers with 0, 3 and 30 μmol g^− 1^ Fe did not lead to a clear change in the beige fiber color of blank-altered fibers, whereas addition of 300 μmol g^− 1^ Fe again changed the color of the fibers to yellow (Additional file 1: Figure S2).

### Coordination environment of ^57^Fe after interaction with chrysotile surfaces

The contribution of the subspectra to the overall Mössbauer spectrum differed per fiber type (Fig. 2; Additional file 1: Table S2), indicating that preconditioning induced a shift in ^57^Fe species distribution. Preconditioning with DFOB reduced the fraction of Fe present as Fe^3+^_tet_ in comparison to the blank-altered treatment (Fig. 2 panel a an b; Additional file 1: Table S2c) and lowered the Fe^3+^_tet_ content by half, from 15.0 to 7.7 μmol g^− 1^ Fe (Additional file 1: Table S7). As only 4% of the fiber mass had dissolved during preconditioning, this suggests that Fe^3+^_tet_ sites are not homogenously distributed over the chrysotile bulk, but are strongly enriched in Si layers near the fiber surface. Addition of 3 μmol of ^57^Fe per gram of DFOB-altered fibers increased the ^57^Fe^3+^_tet_ fraction (Fig. 2 panel a and c) from 2.2 to 3.8% (Additional file 1: Table S2c). This corresponds with an increase in total Fe^3+^_tet_ bulk content by 3% (Additional file 1: Table S7), suggesting a marginal recovery of Fe^3+^_tet_ sites at chrysotile surfaces by addition of ^57^Fe. Most added ^57^Fe (1.8 μmol g^− 1^ Fe ≈ 60%) was retrieved as Fe^3+^_oct_. In contrast, addition of 3 μmol of ^57^Fe per gram of blank-altered fibers resulted in a decrease in ^57^Fe^3+^_tet_ fraction (Fig. 2 panel b and d) from 4.0 to 3.3% (Additional file 1: Table S2c) and a negligible change (0.3%) in total Fe^3+^_tet_ bulk content (14.9 μmol g^− 1^ Fe, Additional file 1: Table S7). Most added ^57^Fe (1.8 μmol g^− 1^ Fe ≈ 60%) was retrieved as magnetite.

### Dissolution of metals and Si from pristine and preconditioned chrysotile fibers in the presence of H_2_O_2_

In Fig. 3, Mg and Si concentrations mobilized from pristine and preconditioned chrysotile fibers in the presence of H_2_O_2_ (initial concentration: 3.34 g L^− 1^) are reported as a function of time. Fe concentrations were in the sub-micromolar range throughout all these experiments (data no shown). In contrast to our results, Ghio et al. (1998) reported somewhat higher mobilized Fe concentrations from a different chrysotile preparation by H_2_O_2_ [32].

For Mg mobilization from pristine chrysotile fibers, two stages could be distinguished (Fig. 3, Panel a1): a fast first stage during which the outermost Mg layer of the fibers rapidly dissolved (first few days), and a slower second stage during which the outermost Mg layer had been completely dissolved, Si dissolution had become rate limiting and mobilized Mg concentrations reached a plateau at approximately 500 to 600 μmol L^− 1^ (between 96 and 336 h). Mg mobilization from DFOB-altered and blank-altered fibers could not be divided into two dissolution stages, but increased linearly throughout the experiment. Si concentrations mobilized from all three fiber types increased linearly throughout the experiment (Fig. 3, Panel a2). Mobilized Si concentrations were consistently higher for DFOB-altered fibers than for blank-altered and pristine fibers. Adsorption and precipitation of added Fe onto DFOB-altered fiber surfaces decreased the rate of Mg (Fig. 3, Panel b1) and Si (Fig. 3, Panel b2) mobilization throughout the experiment. The decrease in Mg and Si dissolution rates were however not proportional to the amount of Fe applied and reached a maximum of approximately 50% with addition of 30 μmol g^− 1^ Fe. Adsorption and precipitation of added Fe onto blank-altered fiber surfaces did not decrease Mg and Si mobilization as strongly as for DFOB-altered fibers (Fig. 3, Panel c1 and c2, respectively): addition of 300 μmol g^− 1^ Fe only decreased mobilized Mg concentrations by 18% and mobilized Si concentrations by 20% after 336 h.

### H_2_O_2_ decomposition by pristine and preconditioned chrysotile fibers

H_2_O_2_ decomposition kinetics in the presence of chrysotile and MOPS buffer could be well described with a first order rate equation in H_2_O_2_ concentration:

1 $$ {Rate}_{\left({H}_2{O}_2\right)}=-\frac{d\left[{H}_2{O}_2\right]}{dt}={k}_{tot}\left[{H}_2{O}_2\right] $$

in which k_tot_ is the overall decomposition constant. Chrysotile fibers accelerated H_2_O_2_ decomposition relative to the MOPS-buffer control treatment by a factor 2 to 5, depending on the pretreatment (Fig. 4a, Table 2). H_2_O_2_ decomposition was fastest with pristine fibers and slowest with DFOB-altered fibers. The addition of DFOB as a redox quencher for Fe largely inhibited differences in the H_2_O_2_ decomposition rate between pristine, blank-altered and DFOB-altered fibers (Fig. 4b). In the treatment with DFOB-altered fibers, the application of DFOB as redox quencher had no effect on H_2_O_2_ decomposition; for the treatments with pristine and blank-altered fibers, H_2_O_2_ decomposition decreased as a result of DFOB addition (Fig. 4a and b). For the treatments with DFOB-altered fibers and DFOB-altered fibers + 0 μmol g^− 1^ Fe, k_tot_ values did not differ (2.5*10^− 3^ h^− 1^), demonstrating that the fiber preparation procedure without Fe addition did not affect the H_2_O_2_ decomposition rate. Fe addition to DFOB-altered fibers increased k_tot_ values by up to over a factor 2 in the DFOB-altered fibers + 300 μmol g^− 1^ Fe (5.3*10^− 3^ h^− 1^); the increase in k_tot_ was non-proportional to the amount of Fe added (Fig. 4c, Table 2). A non-proportional increase in k_tot_ values was also found for treatments in which Fe had been added to blank-altered fibers. The relative increase was however smaller, amounting a factor 1.4 (from 4.2*10^− 3^ h^− 1^ to 6.0*10^− 3^ h^− 1^; Fig. 4d, Table 2).

**Table 2:** H_2_O_2_ decomposition rate constants (k_tot_) and corresponding half-life times determined by fitting the H_2_O_2_ concentration data presented in Fig. 4 to the first order rate equation: ${Rate}_{\left({H}_2{O}_2\right)}=-\frac{d\left[{H}_2{O}_2\right]}{dt}={k}_{tot}\left[{H}_2{O}_2\right]$

Experiment Nr.	Treatment	First order rate constant k_tot_ [h^− 1^]	R^2^	t_1/2_ [h]
1	MOPS pristine fibers	6.2*10^−3^	0.998	112
2	MOPS blank-altered fibers	4.2*10^−3^	0.996	165
3	MOPS DFOB-altered fibers	2.5*10^−3^	0.956	277
4	MOPS DFOB-altered fibers + 0 μmol g^− 1^ Fe	2.5*10^− 3^	0.944	277
5	MOPS DFOB-altered fibers + 3 μmol g^− 1^ Fe	2.7*10^− 3^	0.992	257
6	MOPS DFOB-altered fibers + 30 μmol g^− 1^ Fe	3.5*10^− 3^	0.982	198
7	MOPS DFOB-altered fibers + 300 μmol g^− 1^ Fe	5.3*10^− 3^	0.986	131
8	MOPS buffer *(no fibers)* panel a & c of Fig. 4	1.2*10^− 3^	0.931	578
9	MOPS blank-altered fibers + 0 μmol g^− 1^ Fe	4.2*10^− 3^	0.979	165
10	MOPS blank-altered fibers + 3 μmol g^− 1^ Fe	4.2*10^− 3^	0.983	165
11	MOPS blank-altered fibers + 30 μmol g^− 1^ Fe	4.9*10^− 3^	0.988	142
12	MOPS blank-altered fibers + 300 μmol g^− 1^ Fe	6.0*10^− 3^	0.995	116
13	MOPS buffer *(no fibers)* panel d of Fig. 4	1.4*10^− 3^	0.995	495
14	NaOH pristine fibers	46.7*10^− 3^	0.936	14.8
15	NaOH blank-altered fibers	41.5*10^−3^	0.995	16.7
16	NaOH *(no fibers)*	1.0*10^−3^	0.772	693

The contributions from different reactive sites on chrysotile surfaces to overall H_2_O_2_ decomposition can be estimated in a tiered approach (Table 3), under the assumption that the various degradation mechanisms are independent, and their decomposition constants add up to the k_tot_ of the reaction. Equation 1 can then be rewritten to equation 2:

2 $$ {Rate}_{\left({H}_2{O}_2\right)}=-\frac{d\left[{H}_2{O}_2\right]}{dt}=\left({k}_1+\dots +{k}_n\right)\left[{H}_2{O}_2\right] $$

in which k_1 to n_ represent the contributions of the individual H_2_O_2_ decomposition pathways to the overall decomposition constant k_tot_. In addition to contributions from tetrahedral Fe and Fe (hydr)oxide precipitates, the difference in decomposition rate between the MOPS buffer control and the DFOB-altered fiber treatment suggests a contribution from a remnant H_2_O_2_ decomposition pathway (Fig. 4b, Table 3). The control treatment with MOPS buffer-only provided the contribution from the MOPS buffer to H_2_O_2_ degradation. The contribution from the remnant decomposition pathway was calculated by subtracting the contribution from the MOPS buffer from the k_tot_ value of the DFOB-altered fiber treatment, under the assumption that DFOB had removed most Fe from the fiber surfaces. For the contribution from Fe (hydr)oxide precipitates to H_2_O_2_ degradation it was assumed that the outer Mg and Si layer contained approximately 30 μmol g^− 1^ Fe (Additional file 1: Table S1, Walter et al. (2019), [21]), that this Fe largely precipitated in the blank treatment as only a small fraction of the Fe is located in the slowly dissolving Si layer (Table 1), and that precipitation of an additional 30 μmol g^− 1^ Fe had the same effect size on the k_tot_ value as the Fe that precipitated from the outer layer. The contribution of Fe (hydr)oxide precipitates to the k_tot_ value can then be calculated by subtracting the k_tot_ value of the blank-altered treatment from the blank-altered + 30 μmol g^− 1^ Fe treatment. Finally, the contribution from tetrahedral Fe was calculated by subtracting the contributions from the MOPS buffer, Fe (hydr)oxide precipitates and the H_2_O_2_ decomposition pathway from the k_tot_ value of the blank treatment (Table 3).

**Table 3:** Contributions from different reactive surface sites and the MOPS buffer to the overall H_2_O_2_ decomposition rate constant (k_tot_) for the blank-altered fiber treatment. The fitted constants (k (Exp.x)) for the treatments reported in Table 2 were used and linear additivity was assumed

Decomposition mode	Experiment Nr.	Determination of the contribution to k_tot_ of blank- altered fibers	k-value [h^−1^]
1.) MOPS buffer	8	k (Exp.8) = k1	k1 = 1.2*10^− 3^
2.) Remnant H_2_O_2_ decomposition	3	k (Exp.3) – k1 = k2	k2 = 1.3*10^− 3^
3.) Secondary Fe precipitates	11, 9	k (Exp.11) – k (Exp.9) = k3	k3 = 0.7*10^− 3^
4.) Tetrahedral Fe	2	k1 + k2 + k3 + k4 = k (Exp.2) = > k (Exp.2) – k1 – k2 – k3 = k4	k4 = 1.0*10^− 3^

Following this approach, the k_tot_ value of the blank-altered fiber treatment (4.2*10^− 3^ h^− 1^; Table 2, treatment 2) was broken down to contributions from the three types of active surface sites and the MOPS buffer (equation 2). The contributions of the active surface sites to k_tot_ were comparable, varying within a factor 2, and also the contribution from the MOPS buffer fell within this range (Table 3).

The solution pH had a strong effect on the H_2_O_2_ decomposition rate: in 0.1 mol L^− 1^ NaOH (pH 12–13) the decomposition rate by pristine and preconditioned fibers was approximately an order of magnitude faster than at pH 7.4 (Table 2).

### Effect of Fe addition to preconditioned chrysotile fibers on HO^•^ generation

Pretreatment of Shijiazhuang chrysotile asbestos decreased the HO^•^ yield relative to pristine fibers to 50 ± 10% for blank-altered fibers and to 9% for DFOB-altered fibers [21]. The HO^•^ yield of blank-altered and DFOB-altered fibers + 0 μmol g^− 1^ Fe (Fig. 5) corresponded with these values. For all treatments with Fe addition to DFOB-altered fibers, the HO^•^ yield was larger than for the + 0 μmol g^− 1^ Fe treatment. The HO^•^ yield increased non-proportionally with the amount of Fe added, from 7% (+ 0 μmol g^− 1^ Fe) to 36% (+ 300 μmol g^− 1^ Fe) (Fig. 5a; Additional file 1: Table S5). Although a factor 10 more Fe had been added in the DFOB-altered + 300 μmol g^− 1^ Fe treatment than was extracted in the DFOB-pretreatment, the HO^•^ yield remained lower than in the blank-altered + 0 μmol g^− 1^ Fe fiber treatment (Fig. 5; Additional file 1: Table S5), suggesting that the HO^•^ yield could be largely, but not fully recovered. The addition of Fe to blank-altered fibers did not consistently increase the HO^•^ yield of chrysotile (Fig. 5b). Furthermore, the HO^•^ yield of 3 mg of 2-line ferrihydrite was 11% (relative to the HO^•^ yield of 11 mg pristine chrysotile fibers). The total amount of Fe in 3 mg of 2-line ferrihydrite (≈1.8 mg Fe) is a thousand times larger than the 1.8 μg Fe on the fiber surface of the aliquots of DFOB-altered fibers + 3 μmol g^− 1^ Fe. Despite this large difference, the increase in HO^•^ yield (an increase from 7 to 17%) due to the 3 μmol g^− 1^ Fe addition was comparable with the overall HO^•^ yield of 3 mg of ferrihydrite (11%).

## Discussion

### Speciation of added Fe and implications for fiber dissolution

Si dissolution from DFOB-altered fibers was over a factor three faster than from pristine fibers, whereas Si dissolution from blank-altered and pristine fibers were comparably fast (Fig. 3, Panel a2). The faster Si mobilization from DFOB-altered fibers is a consequence of the complexation of Fe^3+^_tet_ by DFOB during pretreatment. Presumably this led to the formation of vacancy sites in the Si layer resulting in Si labilization which enhanced Si dissolution rates [21]. Si mobilization from blank-altered fibers was considerably slower, because no Fe^3+^_tet_ had been removed from the Si layers during pretreatment. The larger Mg mobilization rate from DFOB-altered fibers compared to blank-altered fibers presumably resulted from the larger rate-controlling Si mobilization rate, allowing segments of deeper Mg layers to dissolve more rapidly; in both treatments the outer Mg layer had been dissolved during pretreatment.

Mössbauer spectroscopy analyses of DFOB-altered and blank-altered fibers + 3 μmol g^− 1 57^Fe demonstrated that the absolute increase in tetrahedrally coordinated ^57^Fe content was more than 5 times larger when added to DFOB-altered fibers compared to blank-altered fibers (Additional file 1: Table S7). However, assuming that no isotope exchange occurred, the data imply that only a small fraction of the Fe^3+^_tet_ sites depleted by DFOB were recovered by ^57^Fe additions.

Despite the apparently low recovery of depleted vacancy sites as observed by Mössbauer spectroscopy, the interaction of Fe with DFOB-altered fibers re-stabilized the labilized Si layer, reduced the Si dissolution rate, and consequently also reduced the Mg dissolution rate (Fig. 3, panel b1 and b2). The 25% reduction in Si and Mg dissolution rate by addition of only 3 μmol g^− 1^ Fe and the fact that Fe addition beyond 30 μmol g^− 1^ did not lead to a further decrease in dissolution rates, supports that the effect of Fe addition originates from the stabilization of the Si layer rather than from surface coverage by precipitated Fe (hydr)oxide minerals that prevent dissolution. The latter observation also suggests that between addition of 3 and 30 μmol g^− 1^ Fe, all vacancy sites became occupied with tetrahedrally coordinated Fe and further Fe addition did not affect dissolution rates. The absence of similar trends in Si and Mg dissolution for Fe addition to blank-altered fibers further indicates that external Fe only becomes tetrahedrally coordinated if there are vacancy sites present in the surface Si layer (Fig. 3, Panel c1 and c2).

### Active sites of H_2_O_2_ decomposition on chrysotile surfaces

At pH 7.4, the H_2_O_2_ decomposition rate (Fig. 4a) (as well as the HO^•^ yield (Fig. 5)) was highest in the treatment with pristine fibers. This is presumably related to a (transient) contribution from Fe in the outermost Mg layer, which dissolves within a few days at this pH. In the treatment with NaOH the Mg layer did not dissolve at all (Additional file 1: Table S8) and the lasting contribution from Fe in this layer may in part explain the higher H_2_O_2_ decomposition rate.

In addition to two Fe-related modes of H_2_O_2_ decomposition by chrysotile, a third, remnant mode was identified (Fig. 4a and b), which, to our knowledge, had not yet been described for asbestos. It made the largest contribution to the k_tot_ of blank-altered fibers in our experiments (Table 3) and may also be relevant in vivo. Magnetite impurities in the Shijiazhuang chrysotile that do not dissolve during the DFOB pretreatment may contribute to the remnant H_2_O_2_ decomposition mode. H_2_O_2_ is more rapidly decomposed by magnetite than by asbestos per unit of mass [31], but magnetite is only a phase contaminant in Shijiazhuang chrysotile asbestos (1.5 ± 0.2%), whereas chrysotile is the predominant phase (86.4 ± 4.6%) [21]. Therefore, we assume that the contribution of magnetite to H_2_O_2_ decomposition rates is small. Furthermore, other substituted metal ions (e.g. Cr, Mn, Ni) that are not or only slowly mobilized by DFOB might have contributed to the remnant H_2_O_2_ decomposition mode. And finally, the contribution from small amounts of remnant Fe that were either not mobilized by DFOB during the pretreatment or that became exposed during the H_2_O_2_ decomposition experiments as a result of Mg and Si dissolution is counted towards the remnant decomposition mode.

In spite of the smaller surface concentration of Fe^3+^_tet_ in blank-altered fibers relative to octahedral Fe which had precipitated as Fe (hydr)oxide minerals, their contributions to H_2_O_2_ decomposition were comparable (Table 3). Several factors may contribute to the comparatively large contribution of Fe^3+^_tet_ per mole Fe. First, only a fraction of the Fe in Fe precipitates resides at the mineral surface and is able to react with H_2_O_2_, whereas all tetrahedral Fe substituted into the exposed Si layer can contribute to H_2_O_2_ decomposition. Secondly, in other silicate minerals like nontronites, it has been shown that Fe^3+^_tet_ is preferentially reduced over octahedral Fe [55–57] suggesting a lower redox potential of Fe^3+^_tet_ in silicate minerals. This lower redox potential may contribute to a higher reactivity of Fe^3+^_tet_ with regard to H_2_O_2_ decomposition. Also for Fe (hydr)oxide minerals, it has been demonstrated that for equal masses the H_2_O_2_ decomposition rates were larger for minerals containing Fe^3+^_tet_, like magnetite (even higher than chrysotile), than for minerals that do not contain Fe^3+^_tet_, like hematite [31].

Similarly to H_2_O_2_ decomposition, a much higher reactivity with respect to the HO^•^ yield was observed for tetrahedral Fe than for octahedral Fe precipitates. The reason for the high redox reactivity of Fe^3+^_tet_ in silicates (and potentially Fe (hydr)oxide minerals) has, to our knowledge, not yet been examined.

### Active sites of HO^•^ generation by structural and external Fe on chrysotile surfaces

Contrary to Fe addition to blank-altered fibers, Fe addition to DFOB-altered fibers clearly increased HO^•^ generation by chrysotile (Fig. 5a). Since DFOB-altered fibers were significantly depleted in Fe^3+^_tet_ sites, this suggests that upon Fe addition, Fe was incorporated into vacancy sites in the Si layer of DFOB-altered fiber surfaces where it became tetrahedrally coordinated and particularly active in HO^•^ generation. It should be noted, however, that Mössbauer data seem to indicate that addition of 3 μmol g^− 1^ ^57^Fe only resulted in a small increase of Fe^3+^_tet_; only 3.0% of the Fe^3+^_tet_ removed in the DFOB pretreatment was recovered through Fe addition. HO^•^ generation, however, recovered to a substantially larger extent by 3 μmol g^− 1^ Fe addition: 10 percentage points relative to untreated fibers, corresponding to 19% of the difference between the blank-altered + 0 μmol g^− 1^ Fe treatment (with the pristine Fe^3+^_tet_ content) and the DFOB-altered + 0 μmol g^− 1^ Fe treatment (with Fe^3+^_tet_ mostly depleted).

The limited recovery of Fe^3+^_tet_ by 3 μmol g^− 1^ ^57^Fe addition may be related to the observed enhanced Si dissolution, possibly creating vacancy sites in the Si layer and subsequent edge pit formation. Edge pit formation would make the sites unsuitable for accommodating Fe^3+^_tet_ coordination when the ^57^Fe was added. However, the factor six discrepancy between recovered Fe^3+^_tet_ and recovered HO^•^ generation seems to indicate that recovered Fe^3+^_tet_ sites may be underestimated by Mössbauer data. Indeed, the recovery of Fe^3+^_tet_ sites was calculated under the assumption that no Fe-isotope exchange occurred in these sites over the timescales of the experiment. Considering the significant time gap between ^57^Fe addition and Mössbauer spectroscopy, it is conceivable that isotope exchange reactions did occur over the timescales of the experiment. In this case, we may have under-estimated the increase of Fe^3+^_tet_ sites after addition of ^57^Fe. Finally, the reactivity of Fe^3+^_tet_ sites regarding HO^•^ generation may be heterogeneous as a result of differences in local coordination environment and the recovery of such sites may not be linearly related to the recovery of reactivity.

Blank-altered fibers do not have vacancy sites in the Si layer, and therefore addition of Fe did not lead to a clear increase in HO^•^ yield. However, the HO^•^ yield of the blank-altered + 0 μmol g^− 1^ Fe treatment, in which surface Fe^3+^_tet_ was preserved, was still higher than the HO^•^ yield of fibers from the DFOB-altered + 300 μmol g^− 1^ Fe treatment. This suggests fewer exposed Fe^3+^_tet_ surface sites in the latter treatment, potentially as a result of a loss of vacancy sites due to ongoing dissolution of the Si layer.

Assuming that Fe addition to blank-altered fibers mainly lead to precipitation of Fe (hydr)oxide minerals, the lack of differences in HO^•^ yield between blank-altered fiber treatments with different amounts of added Fe suggests that these Fe (hydr)oxide minerals do not contribute to HO^•^ generation. This corresponds with results from previous studies: the HO^•^ yield of hematite, which contains no Fe^3+^_tet_ [54], was below the LOD in a study by Fubini et al. (1995) [37], while the HO^•^ yield of magnetite, which does contain structural Fe^3+^_tet_ [54], corresponded with 60% of the HO^•^ yield of chrysotile asbestos on a per mass basis [37]. The difference in reactivity between Fe^3+^_tet_ and octahedral Fe was larger for HO^•^ generation than for H_2_O_2_ decomposition.

The high Fenton reactivity of Fe^3+^_tet_ in chrysotile may, analogously to H_2_O_2_ decomposition, be explained by the lower redox potential of Fe^3+^_tet_ compared to octahedral Fe, as observed in nontronites [55–57], and a potentially rapid back-oxidation of the Fenton-active Fe^2+^_tet_ to Fe^3+^_tet_ by H_2_O_2_, yielding HO^•^. In contrast to Mg and Si mobilization and H_2_O_2_ decomposition, addition of 30 μmol g^− 1^ Fe to DFOB-altered fibers did not recover the HO^•^ yield to the level of blank-altered fibers. For DFOB-altered fibers + 300 μmol g^− 1^ Fe the HO^•^ yield (37 ± 14%) was still significantly lower than for blank-altered fiber + 0 μmol L^− 1^ treatment (58 ± 6%) (Fig. 5, Additional file 1: Table S5). This incomplete recovery of the Fenton reactivity when adding an excess of Fe may suggest a loss of vacancy sites during preconditioning e.g. due to edge pit formation, leading to a smaller number of Fe^3+^_tet_ surface sites than in the blank-altered fiber treatment.

## Conclusions

The results from this study demonstrate that both Fe^3+^_oct_ in Fe (hydr)oxide precipitates and Fe^3+^_tet_ contribute to H_2_O_2_ decomposition by chrysotile asbestos; for asbestos fibers incubated at pH 7.4 in absence of a ligand (blank-altered) the contributions of both Fe species were comparable (within a factor 1.5), despite the excess of octahedral sites. A remnant mode of H_2_O_2_ decomposition by chrysotile was identified, which may be related to magnetite impurities, redox active substituted trace metals not removed by DFOB during pretreatment and remnant Fe. HO^•^ generation by chrysotile asbestos is likely governed by Fe^3+^_tet_; the contribution from Fe precipitates is negligible.

The occurrence of Fe^3+^_tet_ in Fe (hydr)oxide minerals may also be correlated with their HO^•^ yield and their H_2_O_2_ decomposition capacity. However, whereas Fe (hydr)oxide minerals are not pathogenic [58], many silicate minerals other than chrysotile are. In many pathogenic silicates Fe^3+^_tet_ has been detected, e.g. in quartz, in amphiboles and in zeolites [59–64]. Even if these minerals do not contain Fe, our results demonstrate that the presence of vacancy sites in their Si lattice can pose a risk, because incorporation of external Fe into the tetrahedral coordination environment can lead to HO^•^ generation. This may be particularly relevant for zeolites (e.g. erionite), which often have a non-detectable bulk Fe content, but a higher potential to induce mesothelioma than asbestos [2]. The dissolution of tetrahedral Al (which is a stoichiometric constituent of framework silicates) may create abundant vacancy sites in the Si lattice of zeolite fibers, available for the incorporation of Fenton-active tetrahedrally coordinated Fe.

To conclude, our results suggest that Fe^3+^_tet_ governs HO^•^ generation by chrysotile at circumneutral pH, and that Fe^3+^_tet_ may also contribute to the hazard of other pathogenic silicates.

## Supplementary information

**Additional file1: Figure S1.** Wide velocity range Mössbauer spectra of DFOB-altered fibers (Panel a and c) and blank-altered fibers (Panel b and d) with 0 or 3 μmol g^− 1^ added ^57^Fe. **Figure S2.** Preconditioned fibers. Panel a.) From left to right: blank-altered fibers + 0 μmol g^− 1^ Fe, blank-altered fibers + 3 μmol g^− 1^ Fe, blank-altered fibers + 30 μmol g^− 1^ Fe and blank-altered fibers + 300 μmol g^− 1^ Fe; Panel b.) Fiber preparation for Mössbauer analyses, from left to right: blank-altered fibers + 3 μmol g^− 1 57^Fe, blank-altered fibers + 0 μmol g^− 1 57^Fe, DFOB-altered fibers + 0 μmol g^− 1 57^Fe, blank-altered fibers + 3 μmol g^− 1 57^Fe. **Table S1.** Mobilized Mg, Si and Fe concentrations in μmol L^− 1^ during pretreatment (no duplicates available). **Table S2.** Mössbauer hyperfine parameters of DFOB-altered and blank-altered fibers + 0 or 3 μmol g^− 1 57^Fe, analyzed in the narrow (Table a) and the wide (Table b) velocity range. The Fe species distributions were calculated from both the wide and the narrow velocity range data combined (see materials and methods) and are presented in Table c. **Table S3.** Mobilized Mg and Si concentrations from 1 g L^− 1^ pristine, DFOB-altered and blank-altered fibers incubated at pH 7.4 (50 mM MOPS) with addition of 3.34 g L^− 1^ H_2_O_2_. **Table S4.** Residual H_2_O_2_ concentrations during H_2_O_2_ decomposition by pristine fibers, DFOB-altered fibers, blank-altered fibers and the MOPS buffer as a function of time. **Table S5.** HO^•^ yield of DFOB-altered and blank-altered fibers relative to pristine fibers (i.e. 100%). **Table S6.** Results from the statistical analysis of the EPR spin trapping data presented in Fig. 5: *p*-values from the univariate general linear model and Tukey post-hoc test procedure. **Table S7.** Changes in ^57^Fe and total Fe speciation upon addition of 3 μmol g^− 1 57^Fe to blank-altered and DFOB-altered chrysotile at pH 7.4, as determined by Mössbauer spectroscopy. **Table S8.** Mobilized Mg and Si concentrations from 1 g L^− 1^ pristine and blank altered fibers incubated in a 0.1 mol L^− 1^ NaOH solution with addition of 3.34 g L^− 1^ H_2_O_2_.

## Data Availability

The datasets generated and/or analyzed during the current study are available in the supplementary information repository, 10.1186/s12989-019-0333-1. The dataset supporting the conclusions of this article is included within the article (and its additional file).

## Abbreviations

ANOVA: Analysis of variance
BET: Brunauer, Emmet, Teller
DFOB: Desferrioxamine-B
DMPO: 5–5-dimethyl-1-pyrroline N-oxide
DMPO-HO^•^: Adduct of DMPO and HO^•^
DNA: Deoxyribonucleic acid
EPR: Electron paramagnetic resonance
Fe^2+^_oct_: Ferrous octahedral Fe
Fe^2+^_tet_: Ferrous tetrahedral Fe
Fe^3+^_oct_: Ferric octahedral Fe
Fe^3+^_tet_: Ferric tetrahedral Fe
FeDFOB: Fe complexed by DFOB
HEPES: 4-(2-hydroxyethyl)-1-piperazineethanesulfonic acid
ICP-MS: Inductively coupled plasma mass spectrometry
ICP-OES: Inductively coupled plasma optical emission spectrometry
Ipp: Intensity peak-to-peak
LOD: Limit of detection
MOPS: 3-(*N*-morpholino) propanesulfonic-acid
NAA: Neutron activation analysis
nvr: narrow velocity range
PP: Polypropylene
RPM: Rounds per minute
SSA: Specific surface area
Turkeys HSD test: Turkeys honestly significant difference test
UV-VIS: Ultra violet and visible light
WHO-IARC: World health organization, international agency for research on cancer
wvr: wide velocity range
XRD: X-ray diffraction
