# Supplementary material for: Identifying the reactive sites of hydrogen peroxide decomposition and hydroxyl radical formation on chrysotile asbestos surfaces
Source: Part Fibre Toxicol. 2020 Jan 20;17:3. doi: 10.1186/s12989-019-0333-1 (PMC6971994; doi:10.1186/s12989-019-0333-1)
Supplement: Supplementary file 1 — Additional file1: Figure S1. Wide velocity range Mössbauer spectra of DFOB-altered fibers (Panel a and c) and blank-altered fibers (Panel b and d) with 0 or 3 μmol g− 1 added 57Fe. Figure S2. Preconditioned fibers. Panel a.) From left to right: blank-altered fibers + 0 μmol g− 1 Fe, blank-altered fibers + 3 μmol g− 1 Fe, blank-altered fibers + 30 μmol g− 1 Fe and blank-altered fibers + 300 μmol g− 1 Fe; Panel b.) Fiber preparation for Mössbauer analyses, from left to right: blank-altered fibers + 3 μmol g− 1 57Fe, blank-altered fibers + 0 μmol g− 1 57Fe, DFOB-altered fibers + 0 μmol g− 1 57Fe, blank-altered fibers + 3 μmol g− 1 57Fe. Table S1. Mobilized Mg, Si and Fe concentrations in μmol L− 1 during pretreatment (no duplicates available). Table S2. Mössbauer hyperfine parameters of DFOB-altered and blank-altered fibers + 0 or 3 μmol g− 1 57Fe, analyzed in the narrow (Table a) and the wide (Table b) velocity range. The Fe species distributions were calculated from both the wide and the narrow velocity range data combined (see materials and methods) and are presented in Table c. Table S3. Mobilized Mg and Si concentrations from 1 g L− 1 pristine, DFOB-altered and blank-altered fibers incubated at pH 7.4 (50 mM MOPS) with addition of 3.34 g L− 1 H2O2. Table S4. Residual H2O2 concentrations during H2O2 decomposition by pristine fibers, DFOB-altered fibers, blank-altered fibers and the MOPS buffer as a function of time. Table S5. HO• yield of DFOB-altered and blank-altered fibers relative to pristine fibers (i.e. 100%). Table S6. Results from the statistical analysis of the EPR spin trapping data presented in Fig. 5: p-values from the univariate general linear model and Tukey post-hoc test procedure. Table S7. Changes in 57Fe and total Fe speciation upon addition of 3 μmol g− 1 57Fe to blank-altered and DFOB-altered chrysotile at pH 7.4, as determined by Mössbauer spectroscopy. Table S8. Mobilized Mg and Si concentrations from 1 g L− 1 pristine and blank altered fibers [file 12989_2019_333_MOESM1_ESM.docx]

Supplementary information:

Identifying of the reactive sites of hydrogen peroxide decomposition and hydroxyl radical formation on chrysotile asbestos surfaces

Authors: Martin Walter ^a^, Walter D.C. Schenkeveld ^a,b*^, Gerald Geroldinger ^c^, Lars Gille ^c^, Michael Reissner ^d^, Stephan M. Kraemer ^a^

^a^ Department of Environmental Geosciences, University of Vienna

Althanstraße 14 (UZA II), 1090 Vienna, Austria

^b^ Copernicus Institute of Sustainable Development, Faculty of Geosciences, Utrecht University, Princetonlaan 8A, 3584 CB Utrecht, the Netherlands

^c^ Institute of Pharmacology and Toxicology, University of Veterinary Medicine, Vienna

Veterinärplatz 1, 1210 Vienna, Austria

^d^ Institute of Solid State Physics, TU Wien

Wiedner Hauptstraße 8-10, 1040 Vienna, Austria

*Corresponding author:

Walter Schenkeveld; Email: walter.schenkeveld@univie.ac.at

|  |   b.) |
| --- | --- |
|  |  |

SI-Figure 1.) Wide velocity range Mössbauer spectra of DFOB-altered fibers (Panel a and c) and blank-altered fibers (Panel b and d) with 0 or 3 µmol g^-1^ added ^57^Fe. Spectra for blank-altered fibers and DFOB altered-fibers + 3 µmol g^-1 57^Fe were fitted with a ferrihydrite sub-spectrum to account for Fe(hydr)oxide precipitation.

a.)


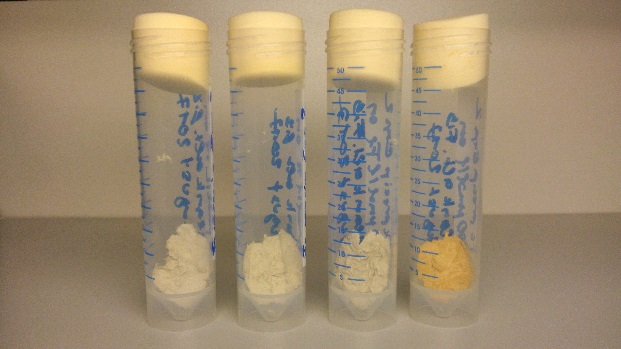

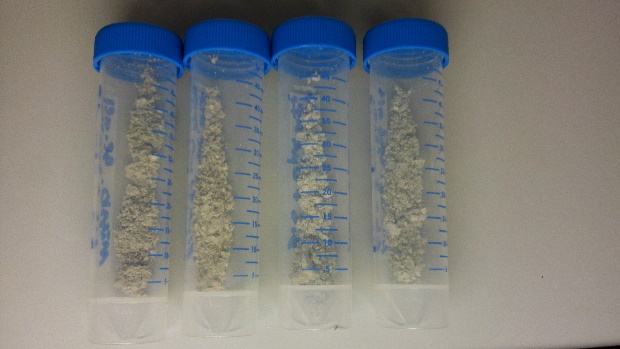


b.)

a.)

b.)

SI-Figure 2.) Preconditioned fibers. Panel a.) From left to right: blank-altered fibers + 0 µmol g^-1^ Fe, blank-altered fibers + 3 µmol g^-1^ Fe, blank-altered fibers + 30 µmol g^-1^ Fe and blank-altered fibers + 300 µmol g^-1^ Fe; Panel b.) Fiber preparation for Mössbauer analyses, from left to right: blank-altered fibers + 3 µmol g^-1^ ^57^Fe, blank-altered fibers + 0 µmol g^-1^ ^57^Fe, DFOB-altered fibers + 0 µmol g^-1^ ^57^Fe, blank-altered fibers + 3 µmol g^-1^ ^57^Fe.

SI-Table 1: Mobilized Mg, Si and Fe concentrations in µmol L^-1^ during pretreatment (no duplicates available). Fibers were pretreated at pH 7.4 without further additions (blank) or with 1 mmol L^-1^ DFOB for 336 h (for H_2_O_2_ decomposition, HO^•^ generation experiments and Mössbauer experiments), or for 24 hours under magnetic stirring with addition of Fe.

| **Fiber pretreatment** | **Mg** | **Si** | **Fe** |
| --- | --- | --- | --- |
|  | **(µmol L^-1^)** | | |
| 336 h pH=7.4 blank pretreatment: H_2_O_2_ decomp + HO^•^ gen Exp. | 530 | 13.2 | 0.0 |
| 336 h pH=7.4 DFOB pretreatment: H_2_O_2_ decomp + HO^•^ gen Exp. | 604 | 80.9 | 28.5 |
| 24 h Fe addition DFOB-alt. fib: H_2_O_2_ decomp + HO^•^ gen Exp. 0 µmol L^-1^ Fe | 97.2 | 54.2 | 0.7 |
| 24 h Fe addition DFOB-alt. fib: H_2_O_2_ decomp + HO^•^ gen Exp. 3 µmol L^-1^ Fe | 62.7 | 53.1 | 0.4 |
| 24 h Fe addition DFOB-alt. fib: H_2_O_2_ decomp + HO^•^ gen Exp. 30 µmol L^-1^ Fe | 54.5 | 27.0 | 0.4 |
| 24 h Fe addition DFOB-alt. fib: H_2_O_2_ decomp + HO^•^ gen Exp. 300 µmol L^-1^ Fe | 86.7 | 46.2 | 0.4 |
| 24 h Fe addition blank-alt. fib: H_2_O_2_ decomp + HO^•^ gen Exp. 0 µmol L^-1^ Fe | 20.5 | 22.5 | 0.0 |
| 24 h Fe addition blank-alt. fib: H_2_O_2_ decomp + HO^•^ gen Exp. 3 µmol L^-1^ Fe | 16.3 | 22.6 | 0.0 |
| 24 h Fe addition blank-alt. fib: H_2_O_2_ decomp + HO^•^ gen Exp. 30 µmol L^-1^ Fe | 39.3 | 43.8 | 0.0 |
| 24 h Fe addition blank-alt. fib: H_2_O_2_ decomp + HO^•^ gen Exp. 300 µmol L^-1^ Fe | 13.4 | 28.2 | 0.0 |
| 336 h pH=7.4 blank pretreatment: Mössbauer Exp. | 533 | 11.3 | 0.1 |
| 336 h pH=7.4 DFOB pretreatment: Mössbauer Exp. | 684 | 84.1 | 31.6 |
| 24 h Fe addition DFOB-alt. fib: Mössbauer Exp. 0 µmol L^-1^ Fe | 111 | 77.6 | 1.1 |
| 24 h Fe addition DFOB-alt. fib: Mössbauer Exp. 3 µmol L^-1^ Fe | 91.4 | 66.9 | 1.5 |
| 24 h Fe addition blank-alt. fib: Mössbauer Exp. 0 µmol L^-1^ Fe | 31.0 | 55.7 | 0.3 |
| 24 h Fe addition blank-alt. fib: Mössbauer Exp. 3 µmol L^-1^ Fe | 28.6 | 16.4 | 0.3 |

SI-Table 2: Mössbauer hyperfine parameters of DFOB-altered and blank-altered fibers + 0 or 3 µmol g^-1 57^Fe, analyzed in the narrow (Table a) and wide (Table b) velocity range. The spectra shown in Figure 2 in the main text are for the narrow velocity range. The narrow velocity range was not large enough for determining the magnetite content. Therefore, magnetite contents were only based on the wide velocity range spectra. B_hf_ refers to the magnetic hyperfine splitting, eQVzz/4 to the quadrupole splitting, CS to the chemical shift rel. to ^57^Co**Rh**, and Γ to the line widths. The Fe species distributions were calculated from both the wide and the narrow velocity range data combined (see materials and methods) and are presented in Table c

Table a: Narrow velocity range:

| Specimen | |  | # | Fe species | B_hf_ | eQV_zz_/4 | CS | Γ/2 | Area |
| --- | --- | --- | --- | --- | --- | --- | --- | --- | --- |
|  |  |  |  |  | mm/s | mm/s | mm/s | mm/s | % |
|  |  |  | 1 | Fe^2+^_oct_ | 0 | 1.181 | 1.172 | 0.191 | 15.0 |
| Blank altered fibers | | | 2 | Fe^3+^_oct_ | 0 | 0.412 | 0.196 | 0.301 | 63.1 |
| 0 µmol g^-1^ ^57^Fe | |  | 3 | Fe^3+^_tet_ | 0 | 0.237 | 0.091 | 0.222 | 4.9 |
| Narrow 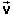 range | |  | 4 | Fe_3_O_4__1 | 15.84 | -0.009 | 0.171 | 0.191 | 4.5 |
|  |  |  | 5 | Fe_3_O_4__1 | 14.62 | 0.052 | 0.53 | 0.225 | 4.7 |
|  |  |  | 6 | Ferrihydrite | 0 | 0.355 | 0.23 | 0.228 | 7.9 |
|  |  |  |  |  |  |  |  |  |  |
|  |  |  | 1 | Fe^2+^_oct_ | 0 | 1.272 | 1.079 | 0.194 | 19.1 |
| Blank altered fibers | | | 2 | Fe^3+^_oct_ | 0 | 0.379 | 0.183 | 0.289 | 54.3 |
| 3 µmol g^-1^ ^57^Fe | |  | 3 | Fe^3+^_tet_ | 0 | 0.21 | 0.089 | 0.218 | 4.4 |
| Narrow 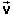 range | |  | 4 | Fe_3_O_4__1 | 15.94 | -0.009 | 0.171 | 0.25 | 7.4 |
|  |  |  | 5 | Fe_3_O_4__2 | 14.77 | 0.052 | 0.53 | 0.28 | 6.7 |
|  |  |  | 6 | Ferrihydrite | 0 | 0.355 | 0.219 | 0.222 | 8.1 |
|  |  |  |  |  |  |  |  |  |  |
|  |  |  | 1 | Fe^2+^_oct_ | 0 | 1.271 | 1.07 | 0.168 | 25.3 |
| DFOB altered fibers | | | 2 | Fe^3+^_oct_ | 0 | 0.385 | 0.172 | 0.317 | 53.4 |
| 0 µmol g^-1^ ^57^Fe | |  | 3 | Fe^3+^_tet_ | 0 | 0.219 | 0.078 | 0.266 | 3.1 |
| Narrow 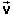 range | |  | 4 | Fe_3_O_4__1 | 15.91 | -0.045 | 0.169 | 0.247 | 10.0 |
|  |  |  | 5 | Fe_3_O_4__2 | 14.71 | 0.028 | 0.516 | 0.248 | 8.2 |
|  |  |  |  |  |  |  |  |  |  |
|  |  |  | 1 | Fe^2+^_oct_ | 0 | 1.27 | 1.082 | 0.169 | 15.8 |
| DFOB altered fibers | | | 2 | Fe^3+^_oct_ | 0 | 0.361 | 0.186 | 0.359 | 60.5 |
| 3 µmol g^-1^ ^57^Fe | |  | 3 | Fe^3+^_tet_ | 0 | 0.236 | 0.091 | 0.271 | 5.3 |
| Narrow 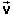 range | |  | 4 | Fe_3_O_4__1 | 15.84 | 0 | 0.167 | 0.24 | 8.1 |
|  |  |  | 5 | Fe_3_O_4__2 | 14.62 | 0 | 0.505 | 0.245 | 7.2 |
|  |  |  | 6 | Ferrihydrite | 0 | 0.347 | 0.231 | 0.223 | 3.1 |

Table b: Wide velocity range:

| Specimen | |  | # | Fe species | B_hf_ | eQV_zz_/4 | CS | Γ/2 | Area |
| --- | --- | --- | --- | --- | --- | --- | --- | --- | --- |
|  |  |  |  |  | mm/s | mm/s | mm/s | mm/s | % |
|  |  |  | 1 | Fe^2+^_oct_ | 0 | 1.28 | 1.02 | 0.10 | 14.2 |
| Blank altered fibers | | | 2 | Fe^3+^_oct_ | 0 | 0.39 | 0.17 | 0.38 | 49.8 |
| 0 µmol g^-1^ ^57^Fe | |  | 3 | Fe^3+^_tet_ | 0 | 0.21 | 0.09 | 0.23 | 3.9 |
| Wide 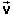 range | |  | 4 | Fe_3_O_4__1 | 15.935 | -0.01 | 0.17 | 0.19 | 13.1 |
|  |  |  | 5 | Fe_3_O_4__1 | 14.773 | 0.05 | 0.52 | 0.23 | 12.3 |
|  |  |  | 6 | Ferrihydrite | 0 | 0.36 | 0.22 | 0.23 | 6.8 |
|  |  |  |  |  |  |  |  |  |  |
|  |  |  | 1 | Fe^2+^_oct_ | 0 | 1.29 | 1.01 | 0.20 | 15.7 |
| Blank altered fibers | | | 2 | Fe^3+^_oct_ | 0 | 0.38 | 0.17 | 0.31 | 40.1 |
| 3 µmol g^-1^ ^57^Fe | |  | 3 | Fe^3+^_tet_ | 0 | 0.20 | 0.09 | 0.23 | 3.3 |
| Wide 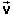 range | |  | 4 | Fe_3_O_4__1 | 15.87 | -0.01 | 0.17 | 0.25 | 19.8 |
|  |  |  | 5 | Fe_3_O_4__2 | 14.8 | 0.05 | 0.53 | 0.27 | 15.1 |
|  |  |  | 6 | Ferrihydrite | 0 | 0.35 | 0.23 | 0.22 | 6.1 |
|  |  |  |  |  |  |  |  |  |  |
|  |  |  | 1 | Fe^2+^_oct_ | 0 | 1.30 | 1.02 | 0.18 | 20.3 |
| DFOB altered fibers | | | 2 | Fe^3+^_oct_ | 0 | 0.38 | 0.17 | 0.33 | 35.3 |
| 0 µmol g^-1^ ^57^Fe | |  | 3 | Fe^3+^_tet_ | 0 | 0.22 | 0.08 | 0.27 | 2.2 |
| Wide 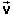 range | |  | 4 | Fe_3_O_4__1 | 15.91 | -0.05 | 0.17 | 0.24 | 25.2 |
|  |  |  | 5 | Fe_3_O_4__2 | 14.71 | 0.03 | 0.52 | 0.25 | 17.2 |
|  |  |  |  |  |  |  |  |  |  |
|  |  |  | 1 | Fe^2+^_oct_ | 0 | 1.29 | 1.08 | 0.18 | 15.1 |
| DFOB altered fibers | | | 2 | Fe^3+^_oct_ | 0 | 0.35 | 0.17 | 0.40 | 41.1 |
| 3 µmol g^-1^ ^57^Fe | |  | 3 | Fe^3+^_tet_ | 0 | 0.22 | 0.09 | 0.28 | 3.7 |
| Wide 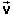 range | |  | 4 | Fe_3_O_4__1 | 15.84 | 0 | 0.17 | 0.24 | 22.1 |
|  |  |  | 5 | Fe_3_O_4__2 | 14.62 | 0 | 0.51 | 0.25 | 16.2 |
|  |  |  | 6 | Ferrihydrite | 0 | 0.35 | 0.23 | 0.22 | 2.0 |

Table c: Calculated ^57^Fe species distribution from the Mössbauer data

|  | blank-altered | blank-altered+ 3 µM ^57^Fe | DFOB-altered | DFOB-altered + 3 µM ^57^Fe |
| --- | --- | --- | --- | --- |
|  | % | % | % | % |
| Fe^2+^_oct_ | 12.3 | 14.5 | 17.8 | 11.5 |
| Fe^3+^_oct_ | 51.8 | 41.1 | 37.6 | 44.1 |
| Fe^3+^_tet_ | 4.0 | 3.3 | 2.2 | 3.8 |
| Magnetite | 25.4 | 34.9 | 42.4 | 38.3 |
| Ferrihydrite | 6.5 | 6.2 | - | 2.3 |

SI-Table 3: Mobilized Mg and Si concentrations from 1 g L^-1^ pristine, DFOB-altered and blank-altered fibers incubated at pH 7.4 (50 mM MOPS) with addition of 3.34 g L^-1^ H_2_O_2_ (Data is plotted in Figure 3 in the main text; SD = standard deviation with n = 2).

Panel a1: Mg.)

| Time | Pristine fibers | SD | Blank-altered fibers | SD | DFOB-altered fibers | SD |
| --- | --- | --- | --- | --- | --- | --- |
| [h] | (µmol L^-1^) |  | (µmol L^-1^) |  | (µmol L^-1^) |  |
| 0,5 | 82.1 | 4.9 | 2.9 | 0.0 | 9.5 | 2.3 |
| 1 | 97.9 | 6.7 | 3.2 | 0.4 | 8.7 | 0.4 |
| 4 | 150 | 14.6 | 4.8 | 0.4 | 15.8 | 0.9 |
| 8 | 199 | 21.9 | 7.9 | 0.6 | 20.0 | 0.8 |
| 24 | 332 | 24.9 | 13.5 | 0.2 | 38.0 | 0.7 |
| 48 | 437 | 25.4 | 20.6 | 0.2 | 51.4 | 0.5 |
| 96 | 511 | 39.3 | 32.9 | 1.4 | 95.9 | 4.7 |
| 168 | 614 | 28.2 | 47.8 | 1.2 | 137 | 4.1 |
| 336 | 602 | 18.9 | 91.3 | 6.6 | 229 | 13.0 |

Panel a2: Si.)

| Time | Pristine fibers | SD | Blank-altered fibers | SD | DFOB-altered fibers | SD |
| --- | --- | --- | --- | --- | --- | --- |
| [h] | (µmol L^-1^) |  | (µmol L^-1^) |  | (µmol L^-1^) |  |
| 0.5 | 6.2 | 0.5 | 4.5 | 0.5 | 6.9 | 0.8 |
| 1 | 5.2 | 0.0 | 3.9 | 0.0 | 5.3 | 0.0 |
| 4 | 6.8 | 0.4 | 3.4 | 0.4 | 6.5 | 0.2 |
| 8 | 6.1 | 1.0 | 3.5 | 1.0 | 9.5 | 0.2 |
| 24 | 7.7 | 0.9 | 6.1 | 0.9 | 17.1 | 0.4 |
| 48 | 8.6 | 0.8 | 8.1 | 0.8 | 25.6 | 1.1 |
| 96 | 13.2 | 1.0 | 13.7 | 1.0 | 46.6 | 1.3 |
| 168 | 14.1 | 0.0 | 16.1 | 0.0 | 72.0 | 2.9 |
| 336 | 22.5 | 1.3 | 35.1 | 1.3 | 117 | 10.0 |

Panel b1: Mg.)

| Time | DFOB-altered fibers  + 0 µmol g⁻¹ Fe | SD | DFOB-altered fibers  + 3 µmol g⁻¹ Fe | SD | DFOB-altered fibers  + 30 µmol g⁻¹ Fe | SD | DFOB-altered fibers  + 300 µmol g⁻¹ Fe | SD |
| --- | --- | --- | --- | --- | --- | --- | --- | --- |
| [h] | (µmol L^-1^) |  | (µmol L^-1^) |  | (µmol L^-1^) |  | (µmol L^-1^) |  |
| 0.5 | 2.9 | 0.2 | 2.7 | 1.1 | 1.0 | 0.2 | 1.9 | 0.1 |
| 1 | 4.6 | 0.5 | 3.4 | 0.5 | 1.4 | 0.2 | 3.3 | 0.4 |
| 4 | 7.9 | 1.0 | 6.1 | 0.6 | 3.6 | 0.3 | 5.4 | 0.3 |
| 8 | 12.6 | 0.4 | 9.4 | 1.2 | 5.1 | 0.4 | 8.9 | 0.5 |
| 24 | 25.7 | 1.0 | 16.7 | 0.2 | 11.3 | 1.1 | 14.7 | 0.2 |
| 48 | 48.9 | 1.0 | 27.6 | 0.4 | 18.2 | 1.4 | 22.6 | 0.2 |
| 96 | 76.1 | 2.1 | 46.4 | 0.1 | 31.6 | 0.8 | 36.6 | 1.0 |
| 168 | 112 | 1.9 | 72.0 | 1.8 | 51.8 | 2.2 | 53.0 | 0.4 |
| 336 | 198 | 1.3 | 151 | 10.8 | 108 | 1.7 | 103 | 1.6 |

Panel b2: Si.)

| Time | DFOB-altered fibers  + 0 µmol g⁻¹ Fe | SD | DFOB-altered fibers  + 3 µmol g⁻¹ Fe | SD | DFOB-altered fibers  + 30 µmol g⁻¹ Fe | SD | DFOB-altered fibers  + 300 µmol g⁻¹ Fe | SD |
| --- | --- | --- | --- | --- | --- | --- | --- | --- |
| [h] | (µmol L^-1^) |  | (µmol L^-1^) |  | (µmol L^-1^) |  | (µmol L^-1^) |  |
| 0.5 | 5.6 | 0.6 | 5.7 | 0.4 | 5.5 | 0.4 | 6.6 | 0.2 |
| 1 | 4.1 | 0.1 | 4.3 | 0.7 | 4.4 | 0.6 | 5.7 | 0.5 |
| 4 | 6.3 | 0.3 | 5.7 | 0.2 | 5.5 | 0.6 | 8.1 | 0.1 |
| 8 | 8.2 | 0.1 | 7.4 | 0.7 | 5.6 | 0.4 | 8.9 | 0.4 |
| 24 | 16.6 | 0.7 | 13.2 | 1.2 | 8.4 | 1.7 | 13.1 | 1.6 |
| 48 | 29.7 | 0.3 | 18.1 | 0.0 | 11.2 | 0.8 | 16.4 | 0.3 |
| 96 | 45.8 | 2.2 | 29.3 | 0.4 | 19.1 | 0.5 | 24.8 | 0.5 |
| 168 | 65.4 | 2.8 | 41.1 | 0.3 | 27.4 | 0.2 | 30.4 | 0.9 |
| 336 | 112 | 0.4 | 81.6 | 5.1 | 53.6 | 0.6 | 53.4 | 2.0 |

Panel c1: Mg.)

| Time | blank-altered fibers  + 0 µmol g⁻¹ Fe | SD | blank-altered fibers  + 3 µmol g⁻¹ Fe | SD | blank-altered fibers  + 30 µmol g⁻¹ Fe | SD | blank-altered fibers  + 300 µmol g⁻¹ Fe | SD |
| --- | --- | --- | --- | --- | --- | --- | --- | --- |
| [h] | (µmol L^-1^) |  | (µmol L^-1^) |  | (µmol L^-1^) |  | (µmol L^-1^) |  |
| 0.5 | 2.4 | 0.4 | 3.2 | 0.2 | 2.2 | 0.1 | 2.1 | 0.0 |
| 1 | 2.6 | 0.3 | 3.3 | 0.1 | 2.3 | 0.3 | 2.2 | 0.3 |
| 4 | 3.3 | 0.7 | 3.8 | 0.1 | 2.9 | 0.6 | 2.7 | 0.2 |
| 8 | 4.2 | 1.1 | 4.5 | 0.2 | 3.6 | 0.0 | 3.4 | 0.1 |
| 24 | 7.1 | 0.5 | 6.7 | 0.5 | 6.3 | 0.5 | 6.0 | 0.4 |
| 48 | 14.3 | 0.6 | 12.2 | 0.9 | 11.6 | 0.2 | 10.0 | 0.5 |
| 96 | 24.8 | 1.4 | 19.9 | 0.1 | 19.7 | 0.9 | 18.2 | 0.8 |
| 168 | 41.0 | 1.8 | 32.1 | 2.8 | 33.5 | 2.9 | 30.0 | 3.3 |
| 336 | 71.6 | 2.4 | 65.0 | 0.9 | 64.0 | 3.9 | 58.8 | 9.8 |

Panel c2: Si.)

| Time | blank-altered fibers  + 0 µmol g⁻¹ Fe | SD | blank-altered fibers  + 3 µmol g⁻¹ Fe | SD | blank-altered fibers  + 30 µmol g⁻¹ Fe | SD | blank-altered fibers  + 300 µmol g⁻¹ Fe | SD |
| --- | --- | --- | --- | --- | --- | --- | --- | --- |
| [h] | (µmol L^-1^) |  | (µmol L^-1^) |  | (µmol L^-1^) |  | (µmol L^-1^) |  |
| 0.5 | 2.4 | 1.4 | 2.9 | 0.3 | 2.3 | 1.4 | 1.2 | 0.2 |
| 1 | 0.9 | 0.2 | 2.0 | 0.1 | 3.1 | 0.2 | 2.5 | 0.1 |
| 4 | 3.0 | 0.4 | 2.0 | 0.8 | 3.1 | 0.6 | 3.8 | 2.1 |
| 8 | 2.1 | 0.8 | 3.4 | 0.3 | 5.4 | 0.0 | 3.0 | 0.2 |
| 24 | 5.1 | 0.6 | 5.5 | 0.1 | 6.7 | 0.5 | 5.1 | 0.8 |
| 48 | 8.4 | 0.5 | 9.7 | 1.3 | 10.7 | 0.5 | 8.3 | 0.8 |
| 96 | 12.9 | 0.0 | 9.9 | 0.1 | 15.3 | 0.4 | 9.7 | 1.2 |
| 168 | 19.1 | 1.8 | 14.9 | 2.0 | 20.0 | 0.9 | 15.0 | 0.7 |
| 336 | 29.5 | 0.0 | 27.7 | 0.2 | 32.0 | 2.4 | 25.2 | 4.6 |

SI-Table 4: Residual H_2_O_2_ concentrations during H_2_O_2_ decomposition by pristine fibers, DFOB-altered fibers, blank-altered fibers and the MOPS buffer as a function of time. The initial H_2_O_2_ concentration was 3.34 g L^-1^. (Data is plotted in Figure 4 in the main text; SD = standard deviation with n = 2).

Panel a.)

| Time | Pristine fibers | | Blank-altered fibers | | DFOB-altered fibers | | MOPS buffer | |
| --- | --- | --- | --- | --- | --- | --- | --- | --- |
| [h] | (g L^-1^) | SD | (g L^-1^) | SD | (g L^-1^) | SD | (g L^-1^) | SD |
| 0.5 | 3.41 | 0.02 | 3.29 | 0.02 | 3.28 | 0.02 | 3.28 | 0.02 |
| 1 | 3.27 | 0.05 | 3.22 | 0.01 | 3.15 | 0.01 | 3.23 | 0.01 |
| 4 | 3.16 | 0.06 | 3.14 | 0.03 | 3.01 | 0.03 | 3.15 | 0.01 |
| 8 | 3.08 | 0.02 | 3.10 | 0.03 | 3.01 | 0.02 | 3.16 | 0.10 |
| 24 | 2.81 | 0.14 | 2.82 | 0.01 | 2.82 | 0.03 | 3.10 | 0.03 |
| 48 | 2.34 | 0.04 | 2.56 | 0.04 | 2.78 | 0.00 | 2.94 | 0.06 |
| 96 | 1.84 | 0.05 | 2.09 | 0.00 | 2.50 | 0.02 | 2.90 | 0.03 |
| 168 | 1.20 | 0.02 | 1.58 | 0.08 | 2.05 | 0.09 | 2.54 | 0.01 |
| 336 | 0.40 | 0.26 | 0.82 | 0.04 | 1.49 | 0.02 | 2.28 | 0.00 |

Panel b.)

| Time | MOPS buffer + DFOB | | Pristine fibers + DFOB | | Blank-altered fibers + DFOB | | DFOB-altered fibers + DFOB | | |
| --- | --- | --- | --- | --- | --- | --- | --- | --- | --- |
| [h] | (g L^-1^) | SD | (g L^-1^) | SD | (g L^-1^) | SD | (g L^-1^) | | SD |
| 96 | 2.98 | 0.03 | 2.57 | 0.07 | 2.54 | 0.02 | 2.42 | 0.05 | |
| 168 | 2.81 | 0.11 | 2.20 | 0.07 | 2.24 | 0.01 | 2.13 | 0.03 | |
| 336 | 2.62 | 0.02 | 1.65 | 0.03 | 1.73 | 0.07 | 1.84 | 0.05 | |

Panel c.)

| Time | DFOB-altered  + 0 µmol g⁻¹ Fe |  | DFOB-altered  + 3 µmol g⁻¹ Fe |  | DFOB-altered  + 30 µmol g⁻¹ Fe |  | DFOB-altered  + 300 µmol g⁻¹ Fe |  | MOPS buffer |  |
| --- | --- | --- | --- | --- | --- | --- | --- | --- | --- | --- |
| [h] | (g L^-1^) | SD | (g L^-1^) | SD | (g L^-1^) | SD | (g L^-1^) | SD | (g L^-1^) | SD |
| 0.5 | 3.32 | 0.05 | 3.40 | 0.01 | 3.28 | 0.05 | 3.27 | 0.02 | 3.28 | 0.02 |
| 1 | 3.12 | 0.09 | 3.20 | 0.06 | 3.21 | 0.00 | 3.13 | 0.04 | 3.23 | 0.01 |
| 4 | 3.02 | 0.00 | 3.20 | 0.01 | 3.00 | 0.05 | 2.97 | 0.02 | 3.15 | 0.01 |
| 8 | 3.04 | 0.11 | 3.19 | 0.00 | 3.06 | 0.01 | 2.97 | 0.00 | 3.16 | 0.10 |
| 24 | 2.86 | 0.04 | 3.03 | 0.00 | 2.82 | 0.02 | 2.68 | 0.03 | 3.10 | 0.03 |
| 48 | 2.73 | 0.05 | 2.82 | 0.08 | 2.54 | 0.00 | 2.31 | 0.01 | 2.94 | 0.06 |
| 96 | 2.44 | 0.01 | 2.52 | 0.07 | 2.30 | 0.03 | 1.80 | 0.06 | 2.90 | 0.03 |
| 168 | 2.00 | 0.03 | 1.98 | 0.07 | 1.72 | 0.01 | 1.25 | 0.00 | 2.54 | 0.01 |
| 336 | 1.51 | 0.01 | 1.36 | 0.05 | 1.05 | 0.07 | 0.58 | 0.01 | 2.28 | 0.00 |

Panle d.)

| Time | Blank-altered  + 0 µmol g⁻¹ Fe |  | Blank-altered  + 3 µmol g⁻¹ Fe |  | Blank-altered  + 30 µmol g⁻¹ Fe |  | Blank-altered  + 300 µmol g⁻¹ Fe |  | MOPS buffer |  |
| --- | --- | --- | --- | --- | --- | --- | --- | --- | --- | --- |
| [h] | (g L^-1^) | SD | (g L^-1^) | SD | (g L^-1^) | SD | (g L^-1^) | SD | (g L^-1^) | SD |
| 0.5 | 3.24 | 0.10 | 3.18 | 0.02 | 3.15 | 0.04 | 3.14 | 0.01 | 3.21 | 0.05 |
| 1 | 3.21 | 0.02 | 3.21 | 0.02 | 3.19 | 0.04 | 3.21 | 0.02 | 3.30 | 0.04 |
| 4 | 3.02 | 0.07 | 3.01 | 0.01 | 3.01 | 0.02 | 3.98 | 0.01 | 3.11 | 0.04 |
| 8 | 3.00 | 0.04 | 3.01 | 0.04 | 2.97 | 0.00 | 3.01 | 0.06 | 3.15 | 0.03 |
| 24 | 2.77 | 0.05 | 2.78 | 0.01 | 2.68 | 0.02 | 2.65 | 0.07 | 3.07 | 0.06 |
| 48 | 2.43 | 0.02 | 2.41 | 0.03 | 2.36 | 0.05 | 2.26 | 0.07 | 2.89 | 0.04 |
| 96 | 1.88 | 0.06 | 1.89 | 0.03 | 1.78 | 0.00 | 1.65 | 0.05 | 2.64 | 0.03 |
| 168 | 1.47 | 0.03 | 1.52 | 0.07 | 1.38 | 0.11 | 1.14 | 0.14 | 2.52 | 0.02 |
| 336 | 0.84 | 0.03 | 0.82 | 0.02 | 0.65 | 0.11 | 0.45 | 0.12 | 2.03 | 0.02 |

SI-Table 5: HO^●^ yield of DFOB-altered and blank-altered fibers relative to pristine fibers (i.e. 100%). (Data is potted in Figure 5 in the main text; SD = standard deviation with n = 4).

Panel a.)

|  | DFOB-altered  + 0 µmol g⁻¹ Fe (0 µg Fe) | DFOB-altered  + 3 µmol g⁻¹ Fe (1.8 µg Fe) | DFOB-altered + 30 µmol g⁻¹ Fe (18 µg Fe) | DFOB-altered + 300 µmol g⁻¹ Fe (180 µg Fe) | 3 mg ferrihydrite (1.8 mg Fe) |
| --- | --- | --- | --- | --- | --- |
| HO^●^ yield (%) | 6.8 | 16.7 | 24.0 | 36.7 | 11.0 |
| SD (%-point) | 2.7 | 3.7 | 5.2 | 15.2 | 2.9 |

Panel b.)

|  | Blank-altered  + 0 µmol g⁻¹ Fe (0 µg Fe) | Blank-altered  + 3 µmol g⁻¹ Fe (1.8 µg Fe) | Blank-altered + 30 µmol g⁻¹ Fe (18 µg Fe) | Blank-altered 300 µmol g⁻¹ Fe (180 µg Fe) | 3 mg ferrihydrite (1.8 mg Fe) |
| --- | --- | --- | --- | --- | --- |
| HO^●^ yield (%) | 57.8 | 79.6 | 50.0 | 53.3 | 11.0 |
| SD (%-point) | 17.5 | 18.3 | 4.8 | 10.3 | 2.9 |

SI-Table 6: Results from the statistical analysis of the EPR spin trapping data presented in Figure 5:
p-values from the univariate general linear model and Tukey post-hoc test procedure.

|  | DFOB-altered + 0 µmol g^-1^ Fe | DFOB-altered + 3 µmol g^-1^ Fe | DFOB-altered  + 30 µmol g^-1^ Fe | DFOB-altered  + 300 µmol g^-1^ Fe |
| --- | --- | --- | --- | --- |
| Blank-altered + 0 µmol g^-1^ Fe | 0.000 | 0.000 | 0.000 | 0.005 |
| DFOB-altered + 0 µmol g^-1^ Fe | - | 0.009 | 0.000 | 0.000 |
| DFOB-altered + 3 µmol g^-1^ Fe | - | - | 0.248 | 0.001 |
| DFOB-altered + 30 µmol g^-1^ Fe | - | - | - | 0.068 |

SI-Table 7: Changes in ^57^Fe and total Fe speciation upon addition of 3 µmol g^-1^ ^57^Fe to blank-altered and DFOB-altered chrysotile at pH 7.4, as determined by Mössbauer spectroscopy. Percent increase refers to the increase in total Fe in the individual crystallographic positions.

|  | blank-altered | | blank-altered + 3 µM ^57^Fe | | % increase |  | DFOB-altered | | DFOB-altered + 3 µM ^57^Fe | | % increase |
| --- | --- | --- | --- | --- | --- | --- | --- | --- | --- | --- | --- |
|  | ^57^Fe | calc. total Fe | ^57^Fe | calc. total Fe | after addition of |  | ^57^Fe | calc. total Fe | ^57^Fe | calc. total Fe | after addition of |
|  | µmol g^-1^ ^57^Fe | µmol g^-1^ Fe | µmol g^-1^ ^57^Fe | µmol g^-1^ Fe | 3 µM ^57^Fe |  | µmol g^-1^ ^57^Fe | µmol g^-1^ Fe | µmol g^-1^ ^57^Fe | µmol g^-1^ Fe | 3 µM ^57^Fe |
| Fe(II)_oct_ | 1.00 | 45.5 | 1.61 | 46.1 | 1.3 |  | 1.34 | 60.9 | 1.21 | 60.8 | -0.2 |
| Fe(III)_oct_ | 4.21 | 191 | 4.58 | 192 | 0.2 |  | 2.83 | 129 | 4.64 | 130 | 1.4 |
| Fe_tet_ | 0.33 | 14.9 | 0.37 | 14.9 | 0.3 |  | 0.17 | 7.53 | 0.40 | 7.77 | 3.2 |
| Magnetite | 2.06 | 93.8 | 3.88 | 95.6 | 1.9 |  | 3.19 | 145 | 4.03 | 146 | 0.6 |
| Ferrihydrite | 0.53 | 24.0 | 0.69 | 24.1 | 0.7 |  | n.d. | - | 0.24 | - | - |
| Total | 8.13 | 370 | 11.1 | 373 | 0.8 |  | 7.52 | 342 | 10.5 | 345 | 0.9 |

SI-Table 8: Mobilized Mg and Si concentrations from 1 g L^-1^ pristine and blank altered fibers incubated in a 0.1 mol L^-1^ NaOH solution with addition of 3.34 g L^-1^ H_2_O_2_. The corresponding H_2_O_2_ decomposition rates are presented in Table 2 in the main text. (SD = standard deviation with n = 4).

|  | Mg pristine fibers | SD | Mg blank altered fibers | SD | Si pristine fibers | SD | Si blank altered fibers | SD |
| --- | --- | --- | --- | --- | --- | --- | --- | --- |
| [h] | (µmol L^-1^) |  | (µmol L^-1^) |  | (µmol L^-1^) |  | (µmol L^-1^) |  |
| 0.5 | 5.4 | 0.1 | 2.7 | 0.2 | 7.3 | 0.7 | 9.5 | 0.4 |
| 1 | 5.1 | 0.1 | 2.7 | 0.0 | 7.7 | 1.9 | 8.9 | 0.2 |
| 4 | 3.8 | 0.1 | 2.5 | 0.2 | 7.9 | 0.2 | 11.2 | 0.6 |
| 8 | 3.4 | 0.1 | 2.7 | 0.0 | 6.3 | 1.0 | 10.6 | 0.3 |
| 24 | 3.0 | 0.1 | 2.6 | 0.1 | 6.0 | 0.2 | 12.3 | 1.6 |
| 48 | 2.9 | 0.1 | 2.7 | 0.0 | 6.7 | 1.4 | 12.1 | 1.0 |
| 96 | 2.9 | 0.1 | 2.8 | 0.2 | 6.7 | 1.4 | 15.0 | 1.0 |
| 168 | 2.7 | 0.0 | 2.6 | 0.0 | 6.7 | 0.5 | 16.9 | 2.2 |
| 336 | 3.1 | 0.2 | 3.3 | 0.2 | 7.0 | 0.1 | 16.5 | 1.6 |
